# Seasonal Physiological Strategies Reveal Contrasting Host–Symbiont Dynamics Among Dominant Indo‐Pacific Reef‐Building Corals

**DOI:** 10.1002/ece3.74044

**Published:** 2026-07-19

**Authors:** Ariana S. Huffmyer, Emma L. Strand, Serena Hackerott, Kevin H. Wong, Danielle M. Becker, Dennis Conetta, Kristina X. Terpis, Ferdinand Pfab, Juliet M. Wong, Zoe Dellaert, Francis J. Oliaro, Ross Cunning, Jose M. Eirin‐Lopez, Steven B. Roberts, Roger M. Nisbet, Hollie M. Putnam

**Affiliations:** ^1^ University of Rhode Island Kingston Rhode Island USA; ^2^ University of Washington Seattle Washington USA; ^3^ Gloucester Marine Genomics Institute Gloucester Massachusetts USA; ^4^ University of Delaware Newark Delaware USA; ^5^ Florida International University Miami Florida USA; ^6^ Rosenstiel School of Marine Atmospheric and Earth Science University of Miami Miami Florida USA; ^7^ University of California Santa Barbara Santa Barbara California USA; ^8^ Duke University Marine Lab Beaufort North Carolina USA; ^9^ John G. Shedd Aquarium Chicago Illinois USA

**Keywords:** acclimatization, cryptic species, plasticity, resilience, symbiosis, trait‐based ecology

## Abstract

As coral reefs face declines driven by thermal stress and the breakdown of coral symbiosis (i.e., coral bleaching), restoration efforts rely on coral health and resilience rankings. However, seasonal plasticity in symbiosis and metabolism and the presence of cryptic species complicates data interpretation. Quantifying seasonal plasticity in coral physiology and incorporating genetic identification are essential for interpreting and drawing conclusions from trait‐based and fitness‐based analyses. To test the effect of seasonal and site variation on physiology, we sampled three ecologically dominant genera, *Acropora*, *Pocillopora*, and *Porites* across three lagoon sites (*n* = 15 tagged colonies genus^−1^ site^−1^) on the north shore of Moʻorea, French Polynesia in January, March, September, and December of 2020. We identified coral host and intracellular Symbiodiniaceae to the highest taxonomic resolution possible and quantified 13 physiological variables. Genetic analyses identified *A. pulchra* and cryptic lineages in *Pocillopora* (*P. meandrina, P. tuahiniensis*) and *Porites* (*P. evermanni, P. lobata/lutea*). *Acropora pulchra* hosted *Durusdinium trenchii* and *Symbiodinium microadriaticum* . Symbiont communities differed between cryptic congeners, with *P. meandrina* hosting *Cladocopium latusorum* and *P. tuahiniensis* hosting *Cladocopium pacificum*, whereas *P. evermanni* and *P. lobata/lutea* both hosted *Cladocopium* (C15), but each with unique C15 profiles. *Acropora* and *Pocillopora* displayed seasonal cycles of symbiont density and productivity (“boom and bust”) in association with light and temperature, a pattern that may contribute to the greater environmental sensitivity previously reported in these taxa. In contrast, *Porites* exhibited greater symbiont stability, with temperature—rather than light—showing stronger associations with host physiology. Increased host biomass under cooler conditions, which may provide greater energy reserves, could represent one mechanism contributing to the comparatively greater stress tolerance observed in massive *Porites*. Collectively, our findings highlight the importance of integrating baseline physiological measurements with host and symbiont genetics when interpreting coral responses across seasons.

## Graphical abstract

Seasonal changes in coral physiology reveal distinct acclimatization strategies among reef‐building corals. Symbiotic generalists exhibit large seasonal fluctuations driven primarily by symbiont dynamics, whereas symbiotic specialists maintain stable symbiont physiology through stronger host regulation. These contrasting strategies provide a mechanistic framework linking holobiont physiology to coral environmental sensitivity and resistance.

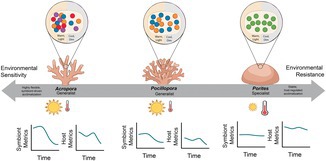

## 1 Introduction

Coral reefs are among the most ecologically diverse ecosystems in the world and at their foundation are coral holobionts: meta‐organisms composed of the coral host, algal symbiont, and associated microbiome (Goulet et al. 2020). These partners exchange crucial nutrients to sustain a mutually beneficial symbiotic relationship (Ainsworth et al. 2010; Roth 2014). However, with ocean waters warming rapidly (Oliver et al. 2021), the maintenance of this symbiosis is threatened (van Woesik et al. 2022), as temperature‐driven metabolic changes destabilize the partnership and increase the risk of dysbiosis, bleaching, and mortality. With increasingly frequent mass coral mortality events (Hughes et al. 2017, 2018), there is an immediate need to understand the mechanisms of coral stress response and acclimatization to environmental change (Putnam 2021). Such shifting baselines in coral physiology (Wall et al. 2021) will impact the range of acclimatization potential possible under future climate change (Grottoli et al. 2014; Logan et al. 2014).

Critical aspects for understanding acclimatization dynamics and organismal stress thresholds include the characterization of the baseline/current physiological state (Cunning and Baker 2013), the breadth of seasonal variation in trait plasticity (Thornhill et al. 2011), and the genetic identity of the holobiont partners (Berkelmans and van Oppen 2006). In particular, there is growing evidence that the multifaceted combination of environmental conditions at any point in the year has the potential to modulate a coral's bleaching susceptibility and response to stress (Holcomb et al. 2012; Scheufen, Krämer, et al. 2017). Such examples demonstrate that both coral and endosymbiont metabolism and physiology are responsive to seasonal environmental change. For example, variation across season is seen in symbiont density (Fitt et al. 2000), tissue biomass (Gómez et al. 2023; Thornhill et al. 2011; Trumbauer et al. 2021; van de Water et al. 2018), skeletal growth (Edmunds and Putnam 2020; Gómez et al. 2023; van de Water et al. 2018), gametogenesis (Keith et al. 2016), lipid content and stable carbon and nitrogen isotopes (Trumbauer et al. 2021), photophysiology (Scheufen, Iglesias‐Prieto, and Enríquez 2017; Trumbauer et al. 2021; Ulstrup et al. 2008; van de Water et al. 2018; Warner et al. 2002), microbial community associations (Miller and Bentlage 2024; Sharp et al. 2017; van de Water et al. 2018), gene expression (Wuitchik et al. 2019), and the epigenome (Hackerott et al. 2023; Rodríguez‐Casariego et al. 2020). Thus, it is critical to investigate the magnitude and mechanisms of these effects across common and dominant reef‐building taxa under natural seasonal variations, especially as novel climate change conditions exacerbate seasonal heatwaves.

Coral function is a product of the tight metabolic interactions between the coral host, its algal intracellular symbionts, and diverse associated microbial communities (i.e., bacteria, fungi, archaea) (Boilard et al. 2020; Li et al. 2023; Rädecker et al. 2015; Siboni et al. 2008; Thompson et al. 2014). As holobionts, reef‐building corals benefit from symbiotic associations with microbial communities, but also contend with the vulnerabilities of their partners. Fast‐growing and weedy species (e.g., *Pocillopora* and *Acropora*) tend to associate with symbionts that support more flexibility and rapid response and productivity while slow‐growing, tolerant species (e.g., *Porites*) tend to associate more with highly specialized and stable partnerships (Putnam et al. 2012). In particular, the nutritional relationship between the coral host and intracellular symbionts (family Symbiodiniaceae; hereafter referred to as “symbionts” or “Symbiodiniaceae”) requires a balance in nutritional cycling to maintain stable relationships, especially under stress (Rädecker et al. 2021, 2023). There is clear evidence that the identity of the Symbiodiniaceae and their photophysiological characteristics (Wall et al. 2020) have functional implications for the holobiont in terms of carbon translocation (Stat et al. 2008), skeletal growth (Little et al. 2004), depth acclimatization (Ziegler et al. 2015), and the sensitivity of the bleaching response (Cunning et al. 2016; Hoadley et al. 2019; Roach et al. 2021). In a notable example, the performance of pairs of the same species of coral ( *Montipora capitata* ) living immediately adjacent to each other is distinctly different when the colonies are dominated by symbionts in the genus *Cladocopium* or *Durusdinium*. Between these holobionts, there are distinct phenotypes of bleaching response (Cunning et al. 2016), carbon translocation from symbiont to host (Allen‐Waller and Barott 2023), metabolomic profiles (Roach et al. 2021), growth (Walker et al. 2025) and bleaching‐induced partial mortality (Matsuda et al. 2020). Therefore, it is critical to evaluate coral holobiont response to environmental variation, considering the identity of both the host and the symbiotic partners (Bellantuono et al. 2012).

Coral reefs in Moʻorea, French Polynesia, the location of our study, experience dynamic environmental conditions, with seasonal and diel temperature changes of ~3°C–4°C ((Edmunds et al. 2010), current study), variable nutrient levels (~1.6 fold seasonal change in nitrogen *Turbinaria* tissue content) (Adam et al. 2021), and spatio‐temporal variation in physical oceanographic dynamics (Edmunds et al. 2010; Leichter et al. 2012). While many coral taxa thrive in these dynamic environments, the genera *Porites*, *Pocillopora*, and *Acropora* have remained dominant in the back reefs (Dahl and Edmunds 2024), each with distinct life history characteristics, symbiotic associations (Putnam et al. 2012), and cryptic species (Burgess et al. 2021; Forsman et al. 2015; Johnston et al. 2018; Rassmussen et al. 2025). *Porites* are mounding corals with thick tissues (Barnes and Lough 1992; Edmunds et al. 2012; Yost et al. 2013) that pass symbionts through vertical transmission (i.e., symbionts are passed to offspring through the oocyte) and are considered environmentally tolerant (Loya et al. 2001; Putnam et al. 2012). *Pocillopora* are branching corals with thin tissues (Yost et al. 2013) that pass symbionts through vertical transmission and exhibit high recruitment rates after a disturbance (Grigg and Maragos 1974; Holbrook et al. 2018) despite their high sensitivity to environmental disturbance (Burgess et al. 2021; Loya et al. 2001). *Acropora* are also branching corals with relatively thin tissues (Bucher and Harrison 2018) that pass symbionts via horizontal transmission and exhibit high sensitivity to environmental change and stress (Loya et al. 2001). Further, *Pocillopora* and *Acropora* spp. (complex clade (Romano and Cairns 2000)) are symbiotic generalists hosting multiple species/strains of *Cladocopium* spp., *Symbiodinium* spp. and/or *Durusdinium* spp. (Putnam et al. 2012). Although *Pocillopora* spp. display high lineage‐specific symbiont specificity (Johnston et al. 2022), the genus as a whole associates with a broader diversity of Symbiodiniaceae taxa than *Porites* (robust clade (Romano and Cairns 2000)), which consistently exhibit high fidelity to *Cladocopium* C15 symbionts (Putnam et al. 2012). Variability in species' symbiotic associations, environmental sensitivity, and tissue and skeletal morphology provide a framework in which to test the dynamics of physiological plasticity across spatial and temporal gradients in important reef‐building corals. Further, understanding cryptic lineage variation within genera is essential because physiological and ecological differences among genetically distinct, but morphologically similar, lineages can influence interpretations of species resilience (Burgess et al. 2021; Forsman et al. 2015; Johnston et al. 2018) and the mechanisms that underpin coral persistence in changing environments.

In light of their different life history strategies and symbiotic associations, we chose these three dominant genera of reef builders (*Porites*, *Pocillopora*, and *Acropora*) to assess holobiont physiological responses across spatiotemporal environmental variation. We conducted extensive physiological sampling of corals from three environmentally variable lagoon sites in Moʻorea, French Polynesia, across four time points in one year (January, March, September, and November 2020). Given the presence of cryptic species in *Acropora* spp. (Rassmussen et al. 2025), *Pocillopora* spp. (Burgess et al. 2021; Johnston et al. 2018), and *Porites* spp. (Forsman et al. 2015), we conducted genetic sequencing and determined that samples collected included *A. pulchra* as well as representatives of *Pocillopora tuahiniensis, Pocillopora meandrina, Porites evermanni*, and *Porites lobata/lutea*. Therefore, we examined the physiological responses to site and seasonal variation, considering cryptic host lineages and their associated symbiont communities.

## 2 Materials and Methods

### 2.1 Replication Statement

**Table:** 

Scale of inference	Scale at which the factor of interest is applied	Number of replicates at the appropriate scale
Coral genera	Genera	Three coral genera: *Acropora*, *Pocillopora*, and *Porites* spp.
Coral species	Species	*Acropora pulchra* (*n* = 42), *Pocillopora tuahiniensis* (*n* = 29), *Pocillopora meandrina* (*n* = 14), *Porites evermanni* (*n* = 33), *Porites lobata/lutea* (*n* = 12)
Reef site	Site	Three reef sites on the north shore of Moʻorea, French Polynesia: Matotia (17.47687203° S 149.805937° W), Vaipahu (17.48137° S 149.84897° W), and Orovau (17.48763° S 149.88742° W)
Seasonal time point	Time point	Four time points: January, March, September, and November 2020
Coral individual	Individual colonies	Fifteen coral individuals in each genus per site

### 2.2 Site and Genus Selection

We tracked physiological traits of three coral genera (*Porites* spp., *Pocillopora* spp., *Acropora* spp.) across spatial and temporal gradients on the north shore backreef of Moʻorea, French Polynesia. Fifteen *Acropora* coral genets were fragmented from colonies on hanging racks in the lagoon on the north shore (17.48393° S, 149.83405° W), individually tagged, and transplanted to each site (*n* = 15 per site) in October 2019. Individual genets of *Pocillopora* and *Porites* were identified and tagged at the three sites in October 2019 (*n* = 15 per genus per site).

**FIGURE 1:**
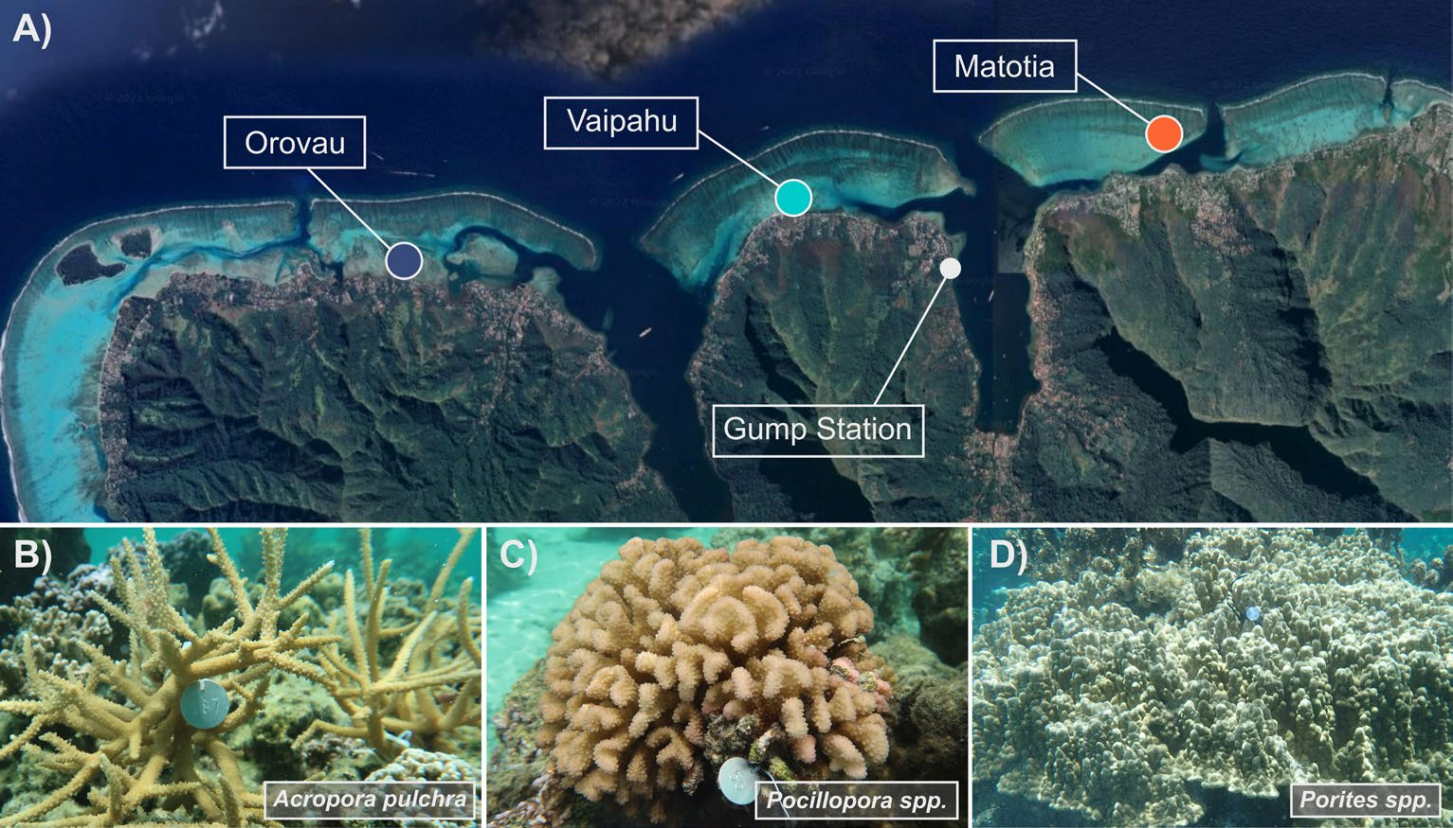
Study design. (A) Three sites (Orovau, Vaipahu, Matotia) were established in October 2019 by transplanting in 15 colonies of (B) *Acropora pulchra* and tagging 15 existing colonies of (C) *Pocillopora* spp. and (D) *Porites* spp. at each site. Colonies were sampled in January, March, September, and November of 2020.

Three study sites were selected across an environmental gradient based on previously characterized nutrient conditions (nitrogen tissue content in *Turbinaria* macroalgae) (Adam et al. 2021). The sites selected were Orovau (relatively lower N:C; 17.48763° S 149.88742° W), Vaipahu (relatively middle N:C; 17.48137° S 149.84897° W), and Matotia (relatively higher N:C; 17.47687203° S 149.805937° W) (Figure 1; site names correspond to the Toponymes de Polynésie Française map from Direction du système d'information de la Polynésie Française). Temperature, pH, and light were measured throughout the study to characterize differences in environmental conditions between these sites (described below). Tagged colonies were sampled at each of the three sites at four time points: January (28 Dec 2019–14 Jan 2020), March (27 Feb–14 March), September (8 Sept–28 Sept), and November (30 Oct–21 Nov) 2020. These sampling time points allowed for *n* = 2 samplings during the wet, humid period (January and March 2020) and *n* = 2 samplings during the cooler, drier season (September and November 2020; (Adam et al. 2021)). Of the 135 total colonies tagged and sampled in January 2020, 112 were sampled in March, 99 in September, and 107 in November. Reduced colony sample size at subsequent time points was due to colony mortality or inability to locate colonies. All experimental and field operations took place at the University of California, Berkeley, Richard B. Gump South Pacific Research Station (UCB Gump Station).

### 2.3 Environmental Characterization

Environmental data loggers were deployed on the reef at each study site in October 2019 and data were collected during each sampling time point. HOBO v2 U22 Temperature loggers (U22‐001; resolution = 0.02°C; accuracy = ±0.21°C) were calibrated with a Digital Traceable Thermometer (4000CC; accuracy = ±0.05°C; resolution = 0.0001°C) prior to deployment. HOBO MX pH loggers (MX2501; accuracy and resolution = ±0.20 mV) were calibrated with pH 7.00 and pH 4.01 NBS standards prior to deployment at each time point. HOBO U24 Conductivity Loggers (U24‐002‐C; resolution = 2 μS/cm) were calibrated with Conductivity Solution RICCA Cat #2248 Conductivity Standard 50,000 μS/cm at 25°C. Odyssey Xtreem PAR loggers were calibrated to a LICOR cosine Underwater Quantum Sensor (LI‐192).

**FIGURE 2:**
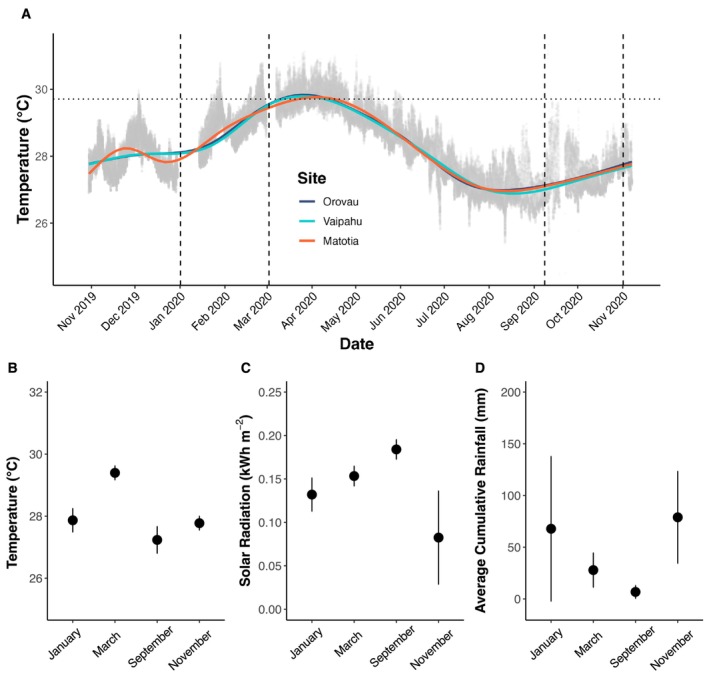
Environmental variables. (A) Temperature (°C) at each site across the one‐year time series study. Generalized additive model lines shown for each site (Orovau = blue; Vaipahu = cyan; Matotia = orange). Temperature observations from data loggers are shown in gray. Dashed lines indicate sampling time points (January, March, September, and November 2020). Dotted line indicates maximum monthly mean calculated from in situ temperature data. (B) Water temperature (°C) as measured in the present study averaged for the 4 weeks prior to sampling time point. (C) Solar radiation (kWh m^−2^) averaged for the 4 weeks prior to sampling time point. (D) Rainfall accumulation (mm) averaged for the 4 weeks prior to sampling time point. Solar radiation and rainfall data obtained from MCR LTER (Washburn and Brooks 2024). All values show mean ± SD.

Due to errors in logging and damaged or missing loggers (when COVID in 2020 reduced our travel capacity to the field), light, pH, and salinity were only available for brief time windows, and therefore we focus primarily on temperature data from the study sites, which are available from the entire time series. Temperature data were collected from November 2019 through November 2020 (Figure 2A). Maximum monthly mean (MMM) temperature was calculated from in situ temperature data. To provide additional environmental context for each sampling time point, we examined publicly available data through the Moʻorea Coral Reef Long Term Ecological Research site (Washburn and Brooks 2024). We visualized data for solar radiation (kWh m^−2^) and cumulative rainfall (mm). These data were collected from one station on the north shore of the island of Moʻorea and therefore we cannot distinguish differences in these characteristics between study sites. For each time point, we calculated the mean (± SD) temperature (Figure 2B), light (Figure 2C) and rainfall (Figure 2D) as the mean of daily values for all observations in the 4 weeks prior to sampling for each time point. Data were not available for the months of February or March 2020 for light or rainfall and therefore we calculated means for the month of April to approximate conditions during the March sampling time point.

### 2.4 Sampling and Live Sample Processing

At each time point, tagged colonies were sampled by clipping three fragments (HMP Convention d'Accueil 2020–2021). Two 2 cm biopsies were preserved for molecular analyses by immediately snap‐freezing in liquid nitrogen on a boat. Samples were returned to University of California Berkeley Richard B. Gump South Pacific Research Station where they were clipped into 1.5 mL tubes with 600 μL of DNA/RNA Shield (Zymo #R1100‐250) followed by storage at −40°C until they were transported to URI for processing (CITES FR2198700194‐E). One 3 cm fragment was collected live from each colony returned to the UCB Gump station and placed in flow‐through seawater tables for 1–6 days during physiological sampling with an equal number of samples per day per genus randomly selected from the water tables for processing. Sample sizes are presented in Table S1. Here, we provide a general description of each metric and refer readers to Supporting Information for further details.

#### 2.4.1 Photosynthesis‐Irradiance (PI) Curves

Photosynthesis‐irradiance (PI) curves were characterized using oxygen production and consumption rates with oxygen measurements of coral fragments under increasing light levels (10 min each at 0 [dark], 18, 68, 113, 169, 243, 499, 709, 844, and 1025 μmol photons m^−2^ s^−1^ photosynthetically active radiation; PAR). Oxygen evolution rates were calculated for each light level interval using localized linear regressions (alpha = 0.2, percentile rank) in the *LoLinR* package (Olito et al. 2017), normalized to surface area, and calculated as μmol O_2_ cm^−2^ h^−1^. PI curves were generated using the quadratic equation (Falkowski and Raven 2013) to calculate maximal photosynthetic rate ($P_{\mathrm{Max}}$), apparent quantum yield (AQY), dark respiration ($R_{D}$), saturating irradiance ($I_{K}$) and compensation irradiance ($I_{C}$) for each colony at each time point. See Supporting Information for details.

#### 2.4.2 Instantaneous Calcification Rates

Instantaneous calcification rate for each fragment was measured using the total alkalinity (TA) anomaly technique (Chisholm and Gattuso 1991). Fragments were incubated for 90 min at 28°C and 500 μmol photons m^−2^ s^−1^ PAR (above saturating irradiance; see Supporting Information) with water samples collected from the initial water source and following the incubation (*n* = 8 coral and *n* = 2 blank samples) and preserved with 75 μL of 50% saturated mercuric chloride (HgCl_2_) solution. Total alkalinity was measured by following the open cell potentiometric titration (SOP3b; (Dickson et al. 2007)). See Supporting Information for details. TA values were normalized to salinity, blank corrected, and normalized to surface area to calculate calcification rates as μmol CaCO_3_ cm^−2^ h^−1^. Following incubation, samples were flash frozen in liquid nitrogen for physiological analyses.

#### 2.4.3 Physiology Sample Processing

Coral tissue was separated from the skeleton using an Iwata Eclipse HP‐BCS airbrush with cold 1× Phosphate Buffer Saline (PBS) solution, resulting in a tissue slurry. Tissue slurry was homogenized using a sterilized PRO Scientific Bio‐Gen PRO200 Homogenizer. Two 1 mL aliquots of tissue homogenate were centrifuged at 13,000 rpm for 3 min (Eppendorf Centrifuge 5415D). The resulting symbiont cell pellet from one aliquot was stored at −40°C for symbiont biomass analyses. The host coral supernatant of the second aliquot was removed and stored at −40°C for host biomass, protein, and antioxidant analyses. The remaining algal pellet in the second aliquot was resuspended in 1 mL of ice‐cold 1× PBS. 500 μL of the resuspended pellet were stored in a −40°C freezer until processing for symbiont density counts and another 500 μL were stored at −40°C for chlorophyll concentration determination. The airbrushed coral skeletons were placed in a drying oven (Fisher Scientific Isotemp Oven) at 60°C for 4 h.

Surface area (cm^2^) was measured on dried skeletons using the wax‐dipping method (Stimson and Kinzie 1991; Veal et al. 2010) and biomass was measured on both host and symbiont fractions as ash‐free dry weight and normalized to fragment surface area (AFDW; mg AFDW cm^−2^). Host soluble protein was quantified using a Bovine Serum Albumin assay (Thermo Scientific Pierce BCA Protein Assay) and normalized to tissue biomass as mg protein mg AFDW^−1^. Antioxidant capacity (TAC) of the host tissue was measured using the Cell BioLabs OxiSelect TAC Assay Kit (Cat # STA‐360) according to the manufacturer's instructions. TAC was normalized to biomass and calculated as μmol Copper Reducing Equivalents (CRE) per mg AFDW.

For chlorophyll quantification, a 500 μL aliquot was centrifuged at 13,000 rpm for 3 min (Eppendorf Centrifuge 5415D), the supernatant removed, and 1 mL of 100% acetone added for 24 h. Symbiont chlorophyll concentration (*a* and *c*_*2*_ pigments) was quantified via spectroscopy with blank subtraction of 750 nm, and using absorbance at 663 nm and 630 nm, following equation 3 for dinoflagellates in 100% acetone from (Jeffrey and Humphrey 1975), with correction for path length. Chl *a* and *c*_*2*_ pigments were normalized to tissue biomass to generate μg pigment mg AFDW^−1^. See Supporting Information for details. The sum of *a* and *c*_*2*_ pigments was calculated to generate total chlorophyll pigment (μg pigment mg AFDW^−1^). Chlorophyll content was also normalized to symbiont cell density to generate μg pigment cell^−1^. Symbiodiniaceae cell density (*n* = 6 replicate counts per sample) of resuspended symbiont pellets was measured using an Improved Neubauer Hemocytometer (Marienfeld Superior, Lauda‐Königshofen, Germany) on a dissecting microscope and was calculated as cells mg AFDW^−1^.

### 2.5 Molecular Characterization

We conducted genetic identification of host haplotypes in *Pocillopora* spp. and *Porites* spp. *Pocillopora* species were identified by amplifying the mitochondrial open reading frame (mtORF) region as described by (Burgess et al. 2021; Johnston et al. 2018) using primers from (Flot et al. 2008) followed by PocHistone 3 region to distinguish species for mtORF haplotype 1a ( *P. meandrina* and *P. eydouxi* ) as described in (Johnston et al. 2018). *Porites* species were identified using the coral nuclear histone region spanning H2A to H4 (i.e., H2) (Tisthammer et al. 2020). *Acropora* samples were identified by amplifying the mitochondrial control region (CRf, CO3r) using primers from (Vollmer and Palumbi 2002). Sequences were aligned and analyzed using Geneious Alignment in GENEIOUS PRIME 2020.2.4. See Supporting Information for details.

Symbiodiniaceae communities were characterized by metabarcoding of the ITS2 rDNA region (Davies et al. 2022). Symbiodiniaceae ITS2 was amplified using the SYM_VAR primers (Hume et al. 2018) with barcodes and Illumina adapters added following (Kozich et al. 2013). Pooled libraries were sequenced on an Illumina MiSeq using a 500‐cycle v2 reagent kit (250 bp paired‐end reads) and custom sequencing primers to initiate forward, reverse and index reads (Kozich et al. 2013). See Supporting Information for details.

### 2.6 Statistical Analysis

#### 2.6.1 Univariate Analysis

All downstream data analyses were conducted using R Statistical Programming v4.2.2 (R Core Team 2022). Univariate statistical analyses for each response (log‐transformed) were conducted using linear mixed effect models (LMM) within each genus in the *lme4* package (Bates et al. 2015). Time point, site, and their interaction were included as main effects with colony as a random intercept to account for repeated measures. For *Pocillopora* spp. and *Porites* spp., which had multiple host haplotypes detected (i.e., “holobionts”), we used colony nested within holobiont as a random intercept. Main effects were evaluated using Type III ANOVA tests in the *lmerTest* package (Kuznetsova et al. 2015) with post hoc comparisons using the *emmeans* package with the Tukey method for controlling for family‐wise error rate (Lenth 2018). The assumption of residual normality was assessed using quantile‐quantile plots. Significance of random effects was tested using ANOVA‐like tables in the *lmerTest* package (Kuznetsova et al. 2015).

Colony missingness in this study refers to instances where a previously recorded colony was not sampled at a subsequent time point, which may have resulted from tag loss, failure to find the colony in the field, or true loss due to mortality or dislodgement, although the exact mechanism could not be determined from field observations. We evaluated patterns of colony missingness in this study to assess whether missingness introduced systematic bias. We classified the missingness/presence of each colony as a binary response at each time point into one of four categories: never missing, temporary absence that was recovered (e.g., missing in March but present in September–November), permanent loss after respective time point (e.g., missing in March and never recovered). Missingness was then calculated as the proportion of colonies in each category. The effect of species, site, and site within species on missingness categories was then evaluated with Fisher's exact tests. Finally, we performed a permutation multivariate analysis of variance (PERMANOVA) for each genus to evaluate the effect of missingness on baseline physiological responses during the January time point to determine whether there were systematic differences in physiology between colonies that went missing and those that were present throughout the study.

#### 2.6.2 Multivariate Analysis

**FIGURE 3:**
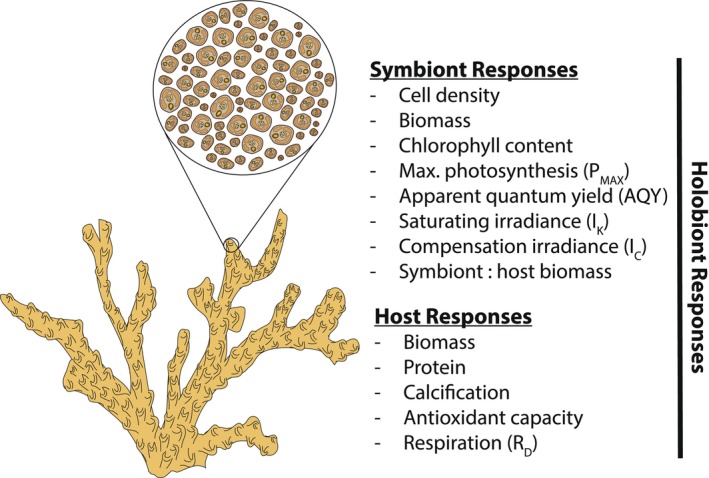
Physiological responses. Physiological responses measured in the host and symbiont in this study.

We used a principal component analysis (PCA) to visualize multivariate physiology using all responses (log‐transformed) for each genus and then for each holobiont within each genus. Differences in multivariate physiology between groups (i.e., genus or holobiont) were tested using a PERMANOVA and permutational multivariate analysis of dispersion (PERMDISP) using euclidean distance in the *vegan* package (Okansen et al. 2017). To assess the assumption of homogeneity of multivariate dispersion, PERMDISP analyses were conducted for each PERMANOVA model. PERMDISP and PERMANOVA results were interpreted together because a significant PERMANOVA result can reflect differences in group centroids, differences in dispersion, or both. Analyses were conducted at the level of combined holobiont responses (all host and symbiont responses) as well as host and symbiont responses (Figure 3). We then conducted multivariate analyses within each genus, including site, time point, and their interaction as main effects and conducted post hoc comparisons using pairwise permanova tests (*pairwiseAdonis* package; (Martinez Arbizu 2017)) and Tukey Honest Significant Difference (HSD) tests (*stats* package; (R Core Team 2022)), respectively. Effect sizes were calculated as Omega‐squared values using the *MicEco* package (Russel 2021) *adonis_OmegaSq* function; (Russel 2021). Within each analysis, the proportion of variance in multivariate physiology explained by each main effect variable (site, time point, and holobiont [cryptic host lineage and associated symbiont community]) individually (i.e., controlling for the influence of the other variable) was quantified with variance partitioning and redundancy analyses (RDA) in the *vegan* package (Okansen et al. 2017). Variance partitioning separated multivariate physiological variation into fractions uniquely attributed to time point, site, holobiont identity, jointly attributed to both site and holobiont identity, and residual variation. For *Porites* spp., holobiont identity and site were partially confounded because *P. lobata/lutea* was absent from Matotia (see Results). Therefore, a portion of variation could not be uniquely assigned to either site or holobiont identity and is instead represented as a shared (e.g., “Site&Holobiont”) fraction, reflecting explanatory variance common to both predictors. To visualize shifts in multivariate physiology across site and time points, we determined multivariate trajectories by calculating the centroid for each time point × site combination and represented the paths between centroids across time as arrows starting at January 2020 and ending at November 2020.

#### 2.6.3 Symbiont Community ITS2 Analysis

Demultiplexed FASTQ files for each sample had the SYM_VAR forward primer prepended to read 1 sequence, and the SYM_VAR reverse primer prepended to read 2 sequences (with dummy quality scores), prior to processing through the SymPortal analysis pipeline (Hume et al. 2019). ITS2 sequences from raw FASTQ files were uploaded and analyzed on the SymPortal remote server (symportal.org; (Hume et al. 2019)). Absolute abundance matrices were analyzed with the *phyloseq* package (v1.44.0; (McMurdie and Holmes 2013)) to calculate relative abundance and visualize ITS2 Type Profile communities (filtered to remove variants with < 1% relative abundance). PERMANOVA statistical tests were performed to assess significant effects of genus, time point, and site on relative abundance of ITS2 profiles using the *vegan* package (Okansen et al. 2017). Due to significant differences in symbiont communities between genera, we further analyzed the effect of holobiont, site, and time point within each genus (holobiont effects only in *Pocillopora* and *Porites*). Due to the significant effect of genus, two‐way ANOVA models were run with site, profile, and holobiont as fixed factors within each genus (holobiont not included for *Acropora*) and post hoc tests in the *emmeans* package with the Tukey method for controlling for family‐wise error rate (Lenth 2018).

We further examined relationships between physiology and variation in the symbiont community through distance‐based redundancy analyses (dbRDA), with symbiont community dissimilarity (Bray–Curtis) as the response variable. For each genus, two separate dbRDAs were conducted including the suite of physiological response metrics (Figure 3) of either the host or the symbiont as predictor variables. Physiological metrics significantly correlated with variation in the symbiont community were identified with ANOVA‐like permutation tests in the *vegan* package (Okansen et al. 2017).

#### 2.6.4 Redundancy Analysis of Effects of Environmental Characteristics on Physiology

Finally, to examine specific seasonal environmental effects on host and symbiont physiology, we conducted redundancy analyses in the *vegan* package (Okansen et al. 2017) to model the effects of light and temperature (described above) on host and symbiont physiological responses. Pearson correlation analyses between the environmental conditions identified that rainfall was correlated (*p* < 0.05) with light and temperature and was therefore removed from further analyses. Maximum and minimum light and temperature were also correlated (*p* < 0.05) with the mean value of each metric, and therefore only mean light and temperature were retained for RDA analyses. Mean light and temperature values for each time point were then modeled against the physiological responses of the host and symbiont separately for *A. pulchra* , *Pocillopora* spp., and *Porites* spp. and visualized as variance explained and in ordination plots.

## 3 Results

### 3.1 *Acropora*, *Pocillopora*, and *Porites* Corals Exhibit Distinct Physiological Characteristics and Cryptic Holobiont Identities

*Acropora* only contained one host species, *Acropora pulchra* (sensu (Conn et al. 2025); Figure S1). *Pocillopora* and *Porites*, on the other hand, each included two host species. *Pocillopora* colonies were identified as either *Pocillopora meandrina* (*n* = 15 total with *n* = 9 at Orovau, *n* = 3 at Vaipahu, and *n* = 3 at Matotia) or *Pocillopora tuahiniensis* (*n* = 29 total with *n* = 5 at Orovau, *n* = 12 at Vaipahu, and *n* = 12 at Matotia; Table S1; Figure S1). *Porites* colonies were identified as either *Porites evermanni* (*n* = 33 total with *n* = 6 at Orovau, *n* = 12 at Vaipahu, and *n* = 15 at Matotia) or in the *Porites lobata/lutea clade* (*n* = 12 total with *n* = 9 at Orovau, *n* = 3 at Vaipahu, and *n* = 0 at Matotia; Table S1; Figure S1).

**FIGURE 4:**
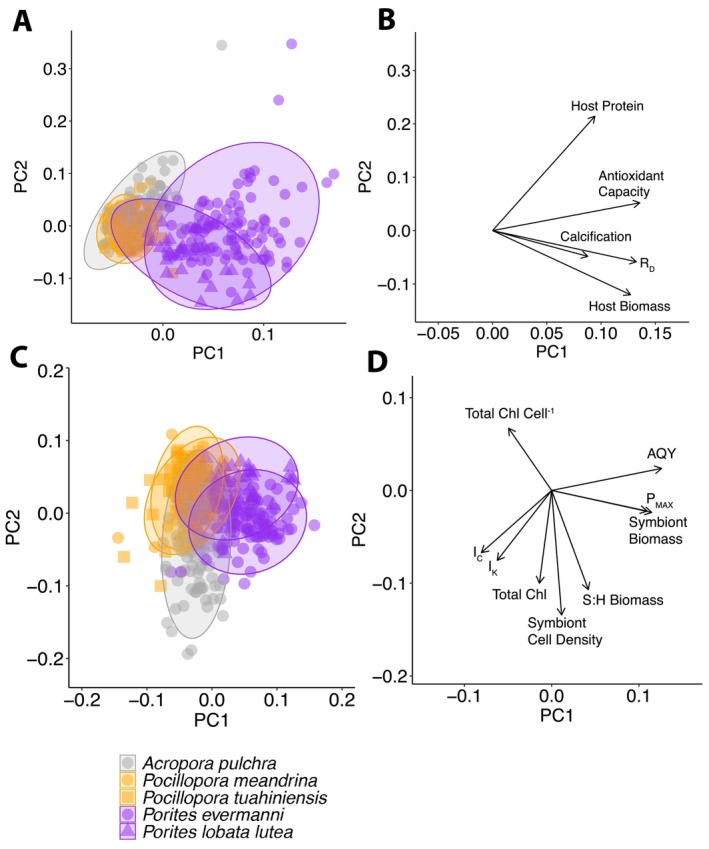
Multivariate physiological responses. (A) Principal components analysis (PCA) of host physiological responses for each holobiont. (B) Biplot of physiological loadings driving separation in multivariate host physiology. (C) PCA of symbiont physiological responses. (D) Biplot of physiological loadings driving separation in multivariate symbiont physiology. Refer to Figure 3 for abbreviations of physiological metrics. Within *Pocillopora* and *Porites*, cryptic species are distinguished by symbol shape to illustrate physiological variation among holobionts. In all plots, *Acropora pulchra* is indicated in gray, *Pocillopora* spp. are in orange ( *P. meandrina* = circles; *P. tuahiniensis* = squares), and *Porites* spp. are in purple ( *P. evermanni* = circles; *P. lobata lutea* = triangles).

Each genus varied in multivariate physiology, with differences reflecting changes in average physiological state and/or differences in variability in both the host (PERMANOVA *p* = 0.001; PERMDISP *p* < 0.001; Figure 4A) and symbiont (PERMANOVA *p* = 0.001; PERMDISP *p* < 0.001; Figure 4C; Table S2). Across genera, differences in host physiology were driven by higher antioxidant capacity, calcification, respiration, and host biomass in *Porites* spp. as well as higher AQY, P_MAX_, and symbiont biomass in *Porites* spp. *Acropora pulchra* exhibited greater symbiont cell density, S:H biomass, and chlorophyll (Figure 4B,D). Multivariate physiology in the host and symbiont were not different between *Pocillopora tuahiniensis* and *Pocillopora meandrina* (Figure 4A,C; Table S3) but symbiont physiology did vary between *Porites evermanni* and *Porites lobata/lutea* (PERMANOVA symbiont *p* = 0.010; host *p* = 0.590; Figure 4A,C; Table S3). These differences were not influenced by multivariate dispersion (PERMDISP *p* > 0.05; Table S3).

The occurrence of missing colonies was higher in *Acropora pulchra* than in the other genera with 36% of originally tagged colonies permanently lost during the course of the study compared to 20% in both *Pocillopora* spp. and *Porites* spp. (Fisher's Exact Test *p* < 0.001). Therefore, further analyses were conducted within each genus to avoid bias introduced by colony missingness. Colony missingness was not influenced by site for any genus (Fisher's Exact Test *p*[*Acropora*] = 0.053, *p*[*Pocillopora*] = 0.156, *p*[*Porites*] = 0.391). Further, baseline multivariate physiology in January did not vary between colonies that were retained throughout the study and those that went missing in *Acropora* (PERMANOVA DF = 1, *R* ^2^ = 0.020, *F* = 0.751, *p* = 0.643), *Pocillopora* (DF = 1, *R* ^2^ = 0.046, *F* = 1.494, *p* = 0.151), nor *Porites* (DF = 1, *R* ^2^ = 0.075, *F* = 2.273, *p* = 0.050). Therefore, there is no evidence that missingness introduced systematic physiological bias in this study.

**FIGURE 5:**
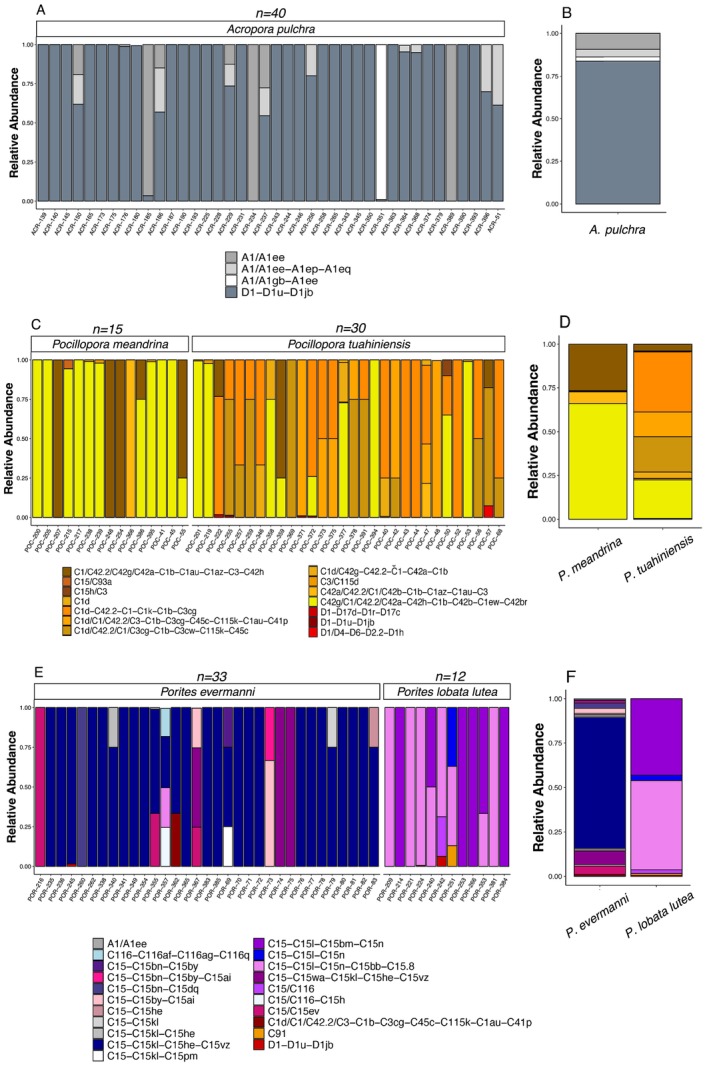
Symbiont community composition. Symbiont ITS2 profile relative abundance for each coral colony in (A) *Acropora pulchra* , (C) *Pocillopora meandrina* and *Pocillopora tuahiniensis*, and (E) *Porites evermanni* and *Porites lobata/lutea*. Mean symbiont ITS2 relative abundance summarized for each holobiont in (B) *Acropora*, (D) *Pocillopora*, and (F) *Porites*. All plots show relative abundance of taxa representing > 1% relative abundance of ITS2 profiles calculated at the colony level. ITS2 profile indicated as the majority profile followed by minor profiles in order of decreasing relative abundance separated by dashes and are shown by color. *n* indicates sample size.

Symbiodiniaceae ITS2 profiles did not change over time and were not variable between sites within each genus (Table S4). *Acropora pulchra* was dominated by the D1‐D1u‐D1jb ITS2 profile (81%) with lesser relative abundance of three A1 profiles (A1/A1ee, A1/A1ee‐A1ep‐A1eq, and A1/A1gb‐A1ee) (Figure 5A,B). There were significant differences in symbiont communities between host haplotypes in *Pocillopora* spp. and *Porites* spp. (PERMANOVA *p* = 0.001; Figure S2) and differences between haplotypes (PERMANOVA *p*[profile:holobiont] < 0.001; Table S5). *Pocillopora meandrina* was associated with *Cladocopium* with 50%–60% relative abundance of C42g/C1/C42.2/C42a‐C42h‐C1b‐C42b‐C1ew‐C42br and lesser component of C1/C42.2/C42g/C42a‐C1b‐C1au‐C1az‐C3‐C42h and C1d/C1/C42.2/C3‐C1b‐C3cg‐C45c‐C115k‐C1au‐C41p (Figure 5C,D). In *P. tuahiniensis*, profile C42g/C1/C42.2/C42a‐C42h‐C1b‐C42b‐C1ew‐C42br had lower relative abundance than in *P. meandrina* and higher presence of C1d‐C42.2‐C1‐C1k‐C1b‐C3cg and C1d/C42.2/C1/C3cg‐C1b‐C3cw‐C115k‐C45c profiles (Figure 5C,D).

*Porites* spp. exhibited clear differences in ITS2 profile relative abundance between host haplotypes. *Porites* spp. showed high fidelity association with *Cladocopium* C15 symbionts but with variation in specific C15 profiles between haplotypes. *Porites evermanni* was associated with a majority of C15‐C15kl‐C15he‐C15vz while *P. lobata/lutea* was associated with C15‐C15l‐C15n‐C15bb‐C15.8 and C15‐C15l‐C15bm‐C15n as major components (Figure 5E,F). Given that each *Pocillopora* and *Porites* haplotype was associated with distinct symbiont communities, we hereafter refer to host and symbiont identity together as distinct “holobionts.” Because there was one host species in *Acropora* ( *Acropora pulchra* ), there is only one holobiont for *Acropora*.

### 3.2 Strong Seasonal Effects on Physiology

**FIGURE 6:**
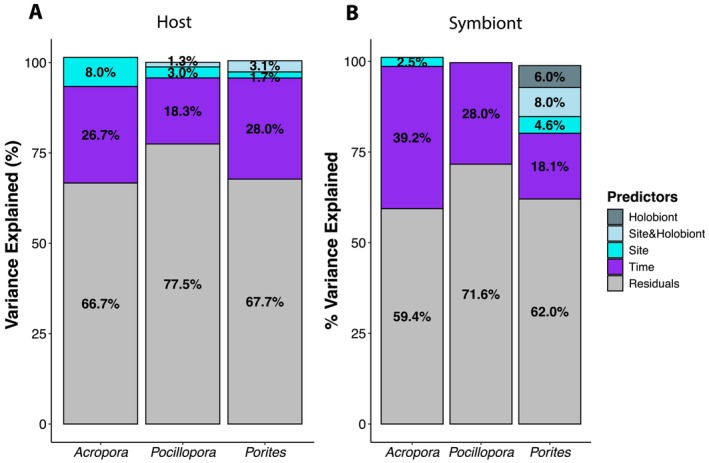
Variance partitioning analysis of effects on physiology. Percent variance explained in multivariate physiology by holobiont (i.e., host species and associated symbiont community; dark gray), site (cyan), time point (purple), and variance that could not be uniquely attributed to site or holobiont identity (light blue) because of partial confounding between factors using redundancy analyses (RDA). Effects shown for host (A), and symbiont responses (B). Only terms explaining > 1% of variance are displayed. Remaining unexplained variation (residuals) is indicated in light gray. Percentages may sum to slightly more than 100% because shared variance among predictor sets contributes to multiple groups and values were rounded for presentation.

We found significant effects of time point, site, and their interaction on multivariate physiology in both host and symbiont responses (further described below). In all three genera and at each biological level, time point explained the highest proportion of variance of any effect (Figure 6, Table S6) with significant site and time interactive effects present in coral physiological responses (Tables S7 and S8).

#### 3.2.1 *Acropora pulchra*

*Acropora pulchra* physiology exhibited significant temporal variation that differed among sites in both host and symbiont physiology (PERMANOVA *p*[time point:site] = 0.033 and 0.021, respectively; Figures S3A and S4A). Significant PERMDISP tests for site (host and symbiont) and time point (symbiont only) indicate that these differences reflect changes in multivariate physiological composition and/or variability among colonies (Tables S9 and S10). Time explained comparatively more variance (26.73%; RDA *p* = 0.001; Figure 6A) and had a larger effect (Omega *R* ^2^ = 0.39) relative to site (Omega *R* ^2^ = 0.09; 8.02% variance explained, RDA *p* = 0.001; Tables S10 and S11). Site and time variation was observed in respiration rates (Linear mixed effect models (LMM) *p*[site] = 0.002; *p*[time point] < 0.001; Figure S5D), host biomass (LMM *p*[site] = 0.033; *p*[time point] = 0.030; Figure S5B), and protein content (LMM *p*[site] = 0.003; *p*[time point] < 0.001; Figure S6C) with seasonal peaks in protein (post hoc *p* < 0.001) and biomass (post hoc *p* = 0.034) in November and highest respiration in January (post hoc *p* < 0.001). Seasonal patterns in antioxidant capacity (LMM *p*[time point:site] = 0.019; Figure S5E) and calcification (LMM *p*[time point:site] < 0.001; Figure S5A) were modulated by site, with antioxidant capacity elevated at Matotia in September (post hoc *p* < 0.05) and calcification peaking in March to September with elevated rates at Orovau (post hoc *p* < 0.001; PC1 in Figure S3D; Table S7).

*Acropora pulchra* symbiont physiology was explained to a similar extent by time (39.24%; RDA *p* = 0.001; Figure 6B), with a small (2.46%), but significant effect of site (RDA *p* = 0.004; Table S11). Time had a larger effect on multivariate physiology (Omega *R* ^2^ = 0.25) than site (Omega *R* ^2^ = 0.07; Table S11). Time significantly influenced photophysiological responses including maximal photosynthesis (LMM *p*[time point] < 0.001; Figure S6C) and saturating irradiance (LMM *p*[time point] < 0.001; Figure S6E), while site modulated seasonal influence on physiological metrics including cell density (*p*[time point:site] = 0.010; Figure S6A), biomass (LMM *p*[time point:site] = 0.047; Figure S6B), chlorophyll (LMM *p*[time point:site] = 0.007; Figure S6G) and S:H biomass (LMM *p*[time point:site] = 0.043; Figure S6I; Table S8). Maximal photosynthesis was elevated in January–March (post hoc *p* < 0.001) with symbiont densities also peaking during this time frame with greater densities at Matotia in the cooler seasonal period (post hoc *p* = 0.024). Symbiont biomass was also elevated in January and was highest during this time point at the Orovau site (post hoc *p* = 0.008). The ratio of symbiont to host biomass (S:H), in contrast, was highest in September (post hoc *p* < 0.001) but was also elevated at Orovau in the warmer months (post hoc *p* = 0.026).

#### 3.2.2 *Pocillopora* spp.

Multivariate physiology in *Pocillopora* spp. varied significantly across time and among sites in both the host (PERMANOVA *p*[time point:site] = 0.001; Figure S3B) and symbiont (PERMANOVA *p*[time point:site] = 0.003; Figure S4B). Significant PERMDISP results for host physiology across time (*p* = 0.042) and for symbiont physiology across both time (*p* < 0.001) and site (*p* = 0.033) indicate that these differences reflect changes in mean multivariate physiology and/or variability (Tables S9 and S10). Site explained a small, but significant, portion of host physiology (3.05%; RDA *p*[site] = 0.002; Figure 6A). Time had a larger effect (Omega *R* ^2^ = 0.24) than site (Omega *R* ^2^ = 0.03). Host responses varied significantly between *P. tuahiniensis* and *P. meandrina* (PERMANOVA *p* = 0.005; PERMDISP *p* = 0.481; Tables S9 and S10; Figure S7), but with a small effect size (Omega *R* ^2^ = 0.02).

*Pocillopora* spp. host respiration and biomass (LMM *p*[time point:site] < 0.001; Table S7; Figure S5) varied seasonally modulated by site. Specifically, corals at Orovau retained lower biomass than those at the other sites in November (post hoc *p* < 0.001; Figure S5B) with respiration higher in September at the Matotia site (post hoc *p* < 0.001; Figure S5D). Time had significant effects on host protein, calcification, and antioxidant capacity (LMM *p*[time point] < 0.001; Table S7; Figure S5). Calcification (Figure S5E) peaked in March–September (post hoc *p* < 0.001; PC2 in Figure S3E), antioxidant capacity was highest in September–November (post hoc *p* < 0.001; Figure S5A; PC1 in Figure S3E), and protein was lowest in November (post hoc *p* < 0.001; Figure S5C; PC1 in Figure S3E). There was only a small effect of haplotype on host physiology in *P. meandrina* and *P. tuahiniensis* (Omega *R* ^2^ = 0.02; Figure S8; Table S12).

Symbiont physiology in *Pocillopora* spp. was significantly explained by time (28.01%; RDA *p* = 0.001; Omega *R* ^2^ = 0.22; Figure 6B; Table S11) and varied significantly between holobionts (PERMANOVA *p* = 0.001; PERMDISP *p* = 0.481; Table S10; Figure S7) but effects were small (Omega *R* ^2^ = 0.04; Table S9). Symbiont biomass (LMM *p* < 0.001) and S:H biomass (LMM *p* < 0.001) were higher in *P. meandrina* than *P. tuahiniensis* (Figure S9; Table S13). Symbiont density was elevated in January and September with lower densities at Orovau (LMM *p*[time point:site] < 0.001; post hoc *p* = 0.007; Table S8; Figure S6A). Symbiont biomass was also lower at Orovau (LMM *p*[site] = 0.033; post hoc *p* = 0.026) and exhibited seasonal peaks in the cooler months of September–November (LMM *p*[time point] < 0.001; post hoc *p* < 0.001; Figure S6B) along with S:H biomass (LMM *p*[time point:site] = 0.016; post hoc *p* < 0.001; Figure S6I). Photosynthesis was highest in the warmer months (LMM *p*[time point] < 0.001; post hoc *p* = 0.004; Figure S6C). I_K_ exhibited a strong peak in March followed by a sharp decrease in September (LMM *p*[time point] < 0.001; post hoc *p* < 0.001; Figure S6E) with AQY reduced during this time point (LMM *p*[time point] < 0.001; post hoc *p* < 0.001; Figure S6D; PC1 in Figure S4E; Table S8).

#### 3.2.3 *Porites* spp.

Physiological responses in *Porites* spp. varied significantly across time and among sites in both host (PERMANOVA *p*[time point:site] = 0.012; Figure S3C) and symbiont physiology (PERMANOVA *p*[time point:site] = 0.001; Figure S4C). PERMDISP tests were not significant for host physiology (*p* > 0.05), whereas significant dispersion among sites for symbiont physiology (*p* < 0.001) indicates that symbiont differences reflect changes in multivariate physiological composition and/or variability (Tables S9 and S10). In the host, time explained 27.98% of physiological variation (RDA *p* = 0.001; Omega *R* ^2^ = 0.24; Table S11) with a small, but significant, amount of variation explained by site (1.69%, RDA *p* = 0.001; Omega *R* ^2^ = 0.04; Figure 6A). Host physiological metrics including antioxidant capacity, biomass, respiration rate, and calcification were higher in *Porites* spp. than *Pocillopora* spp. and *Acropora pulchra* (Figure S5). The effect of holobiont was significant in the host and was due to differences in group means and/or variability (PERMANOVA *p* = 0.001; PERMDISP *p* = 0.035; Tables S9 and S10; Figure S10) explaining 3.09% of variance (Figure 6A). Specifically, antioxidant capacity (LMM *p* < 0.001; Figure S11A) and protein content (LMM *p* < 0.001; Figure S11C) were higher in *P. evermanni* than *P. lobata/lutea* (Table S12). Because *P. lobata/lutea* was not present at Matotia, site and holobiont identity were partially confounded and, therefore, variance partitioning identified shared site‐holobiont fractions (i.e., “Site&Holobiont” in Figure 6) that represents physiological variation explained by both predictors that cannot be assigned to either site or holobiont uniquely.

In the host, antioxidant capacity peaked in September (LMM *p*[time point] < 0.001; post hoc *p* < 0.001; Figure S5A) and respiration rates decreased across the time series (LMM *p*[time point] < 0.001; post hoc *p* < 0.001; Figure S5D; Table S7). Seasonal shifts in host biomass were modulated by site (LMM *p*[time point:site] = 0.028; Figure S5B), with biomass decreasing between January and March at Vaipahu, but remaining stable at the other sites (post hoc *p* = 0.019). Further, site impacted seasonal variation in calcification with lower rates at Vaipahu (LMM *p*[time point:site] = 0.012; post hoc *p* = 0.002; Figure S5E).

Variation in symbiont physiological responses were significantly explained by time (18.12%; RDA *p* = 0.001) and site (4.63%; RDA *p* = 0.001; Figure 6B; Table S11). The effect of holobiont on symbiont physiology was significant (PERMANOVA *p* = 0.001; PERMDISP *p* = 0.097; Figure S10) with holobiont and site:holobiont effects explaining 6.02% and 8.02%, respectively (RDA *p* = 0.001; Figure 6C; Table S11). The effect of holobiont was equally as strong on symbiont physiology as time (Omega *R* ^2^ = 0.16), which were both stronger than the effect of site (Omega *R* ^2^ = 0.05; Table S9). Several symbiont physiological characteristics were higher in *P. evermanni*, including cell density (LMM *p* < 0.001; Figure S12A), symbiont biomass (LMM *p* < 0.001; Figure S12B), I_C_ (LMM *p* = 0.002; Figure S12F), and symbiont:host biomass (LMM *p* < 0.001; Figure S12I). Only cell‐specific chlorophyll content was higher in *P. lobata/lutea* (LMM *p* = 0.024; Figure S12H). Although the mean responses displayed in Figure S6 visually show separation in responses by site, particularly at Matotia (e.g., Figure S6B), this is confounded by the differential presence of holobionts at Matotia and was therefore not statistically significant after accounting for holobiont as a random effect.

Symbiont biomass was stable across seasonal time points (LMM *p*[time point] = 0.071; Figure S6B; Table S8). In contrast, symbiont densities (LMM *p*[time point:site] = 0.008; post hoc *p* = 0.039; Figure S6A), chlorophyll (LMM *p*[time point:site] = 0.038; post hoc *p* < 0.001; Figure S6G), and maximal photosynthesis (LMM *p*[time point] = 0.003; LMM P[site] = 0.022; Figure S6C) were highest in the warmer seasons, particularly at the Matotia site, although site effects are confounded by holobiont identity and therefore cannot be attributed to site alone.

### 3.3 Light and Temperature Changes Across Seasonality Affect Host and Symbiont Physiological Responses

**FIGURE 7:**
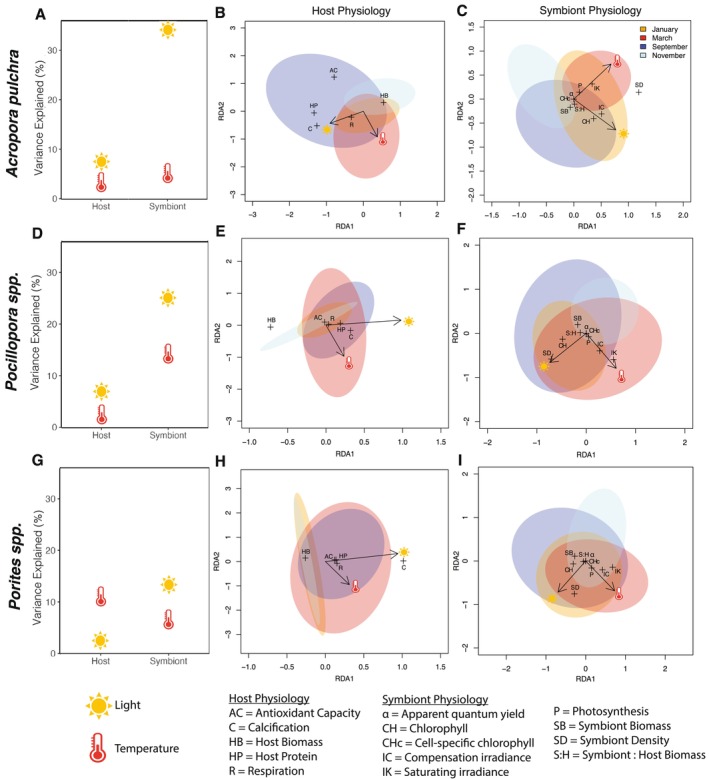
Variance partitioning analysis of temperature and light seasonal effects on physiology. Percent variance explained by light (sun icon) and temperature (thermometer icon) in multivariate physiology of the host and symbiont (x‐axis) for each genus (A = *Acropora pulchra* , D = *Pocillopora* spp., G = *Porites* spp.) as analyzed using redundancy analyses (RDA). Light and temperature effects on host and symbiont physiology were significant for all genera at *p* < 0.05. RDA plots showing how light (sun icon and associated arrow) and temperature (thermometer icon with associated arrow) drive host (B, E, H) and symbiont (C, F, I) physiology for each genus. + indicates the location in the ordination and therefore association of each physiological metric with light and temperature drivers with metrics closer to the arrows being more strongly associated with the respective environmental variable. Metrics in opposite directions of variable arrows are negatively associated and metrics in the same direction as the arrows are positively associated. Metrics that are further from the plot origin have a stronger response to one or more environmental variables while those near the plot origin have weak associations. Longer environmental variable arrows indicate the strength of correlations with associated physiological metrics. Ellipses represent 95% confidence intervals (orange = January, red = March, dark blue = September, light blue = November).

Physiological responses in the host and symbiont were significantly influenced by both light and temperature (RDA *p* < 0.01; Table S14). Across all genera, light explained more variance in symbiont physiology than temperature, with the highest variance explained (34.3%) in *A. pulchra* (Figure 7A). Calcification and host protein content were positively correlated with light and increased during high light seasons, whereas biomass was negatively correlated and was higher in the cooler seasonal period (i.e., biomass in opposite direction from light arrows in Figure 7B,E,H). *Pocillopora* spp. symbiont physiology was also highly responsive to light (25.1%; Figure 7D) with *Porites* spp. symbiont physiology less responsive than the other two genera (13.1%; Figure 7G).

Temperature explained the most variance in symbiont physiology in *Pocillopora* spp. (13.3%; Figure 7D) as compared to < 10% variance explained in *Porites* spp. and *A. pulchra* (Figure 7A,G), driven by positive correlations of photosynthetic parameters I_K_ and I_C_ with temperature (Figure 7F). Host physiology in all species was less responsive to light (2.4%–7.5% variance explained; Figure 7A,D,G) with calcification being the primary light‐driven response in the host (Figure 7B,E,H). Variance in *Porites* spp. host physiology was explained by temperature (10.0%) to a greater extent than *Pocillopora* spp. (1.6%) or *A. pulchra* (2.3%), which was driven by the positive relationship between temperature with respiration and a negative relationship between temperature and host biomass (Figure 7H).

### 3.4 Variation in Symbiont Community Composition Is Associated With Variation in Physiological Responses in *Pocillopora* spp. and *Porites* spp.

Variation in the ITS2 symbiont community composition of *A. pulchra* was not significantly related to either the host (dbRDA *p* = 0.751) or symbiont physiology (dbRDA *p* = 0.624; Table S15). However, variation in the symbiont community composition was related to the physiology of *Pocillopora* spp. and *Porites* spp. In *Pocillopora* spp., the symbiont community composition was significantly correlated with symbiont physiology (dbRDA *p* = 0.001), but there was no significant relationship with host physiology (dbRDA *p* = 0.886; Table S15). Symbiont metrics significantly related to community composition in *Pocillopora* spp. included symbiont biomass (dbRDA *p* = 0.014), total chlorophyll (dbRDA *p* = 0.001), cell‐specific chlorophyll (dbRDA *p* = 0.046), I_C_ (dbRDA *p* = 0.024), and S:H biomass (dbRDA *p* = 0.033; Table S16). The multivariate physiology of the symbiont constrained 14.09% of the variance in the symbiont community composition in *Pocillopora* spp. with the model explaining 7.42% (Adj. *R* ^2^) of the variance (Table S15).

In contrast, variation in the symbiont community composition in *Porites* spp. was related to the physiology of both the host and the symbiont (dbRDA *p*[host] = 0.001, *p*[symbiont] = 0.001; Table S15). The multivariate physiology of the host constrained 14.17% of the variance in the symbiont community composition (model Adj. *R* ^2^ = 10.84%, Table S15), driven by significant correlations with host antioxidant capacity (dbRDA *p* = 0.001) and biomass (dbRDA *p* = 0.002; Table S16). The physiology of the symbiont constrained comparatively more of the variance in the symbiont community composition (22.58%, Adj. *R* ^2^ = 17.01%, Table S15). Symbiont cell density (dbRDA *p* = 0.002), symbiont biomass (dbRDA *p* = 0.001), P_MAX_ (dbRDA *p* = 0.038), and AQY (dbRDA *p* = 0.002) were significantly correlated with symbiont community composition (Table S16).

## 4 Discussion

**FIGURE 8:**
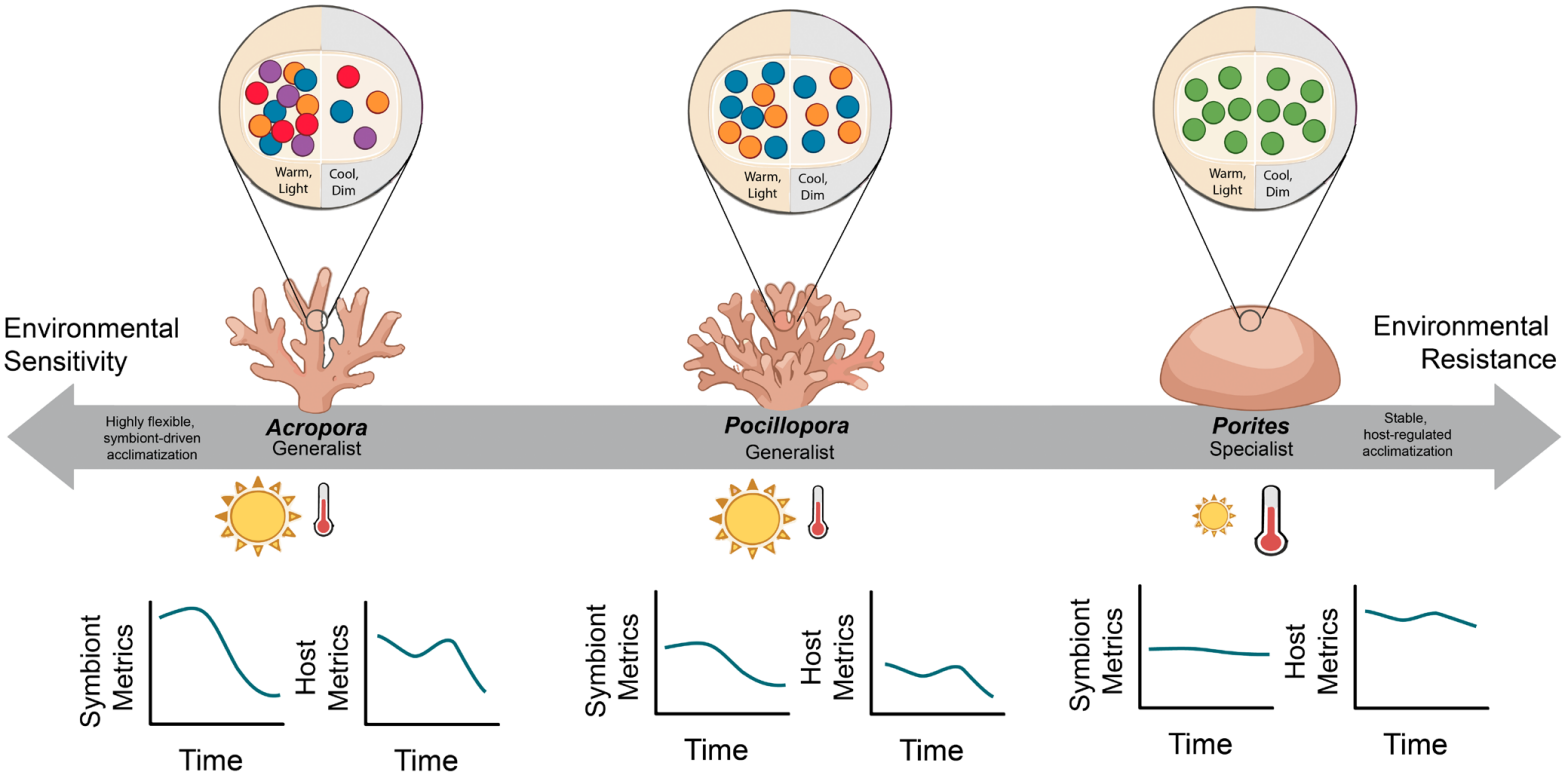
Overview of seasonal acclimatization strategies across dominant Indo‐Pacific coral genera. Seasonal acclimatization strategies in environmentally sensitive, symbiotic generalist corals (*Acropora* and *Pocillopora*) and environmentally resistant, symbiotic specialist corals (*Porites*). *Acropora* hosts the greatest diversity of symbiont taxa (indicated by colored circles representing symbionts) and exhibits the largest seasonal (“warm, light” and “cool, dim” seasonal periods) fluctuations in symbiont physiology, consistent with a symbiont‐driven “boom‐and‐bust” strategy in which changes in symbiont metrics are driven primarily by seasonal variation in light (sun icon) and secondarily by temperature (thermometer icon). *Pocillopora* represents an intermediate strategy, with moderate symbiont diversity and physiological variability. In contrast, *Porites* associates with specialized symbiont communities and maintains relatively stable symbiont physiology across seasons, while host responses are more strongly associated with seasonal temperature than light. These patterns support a continuum from flexible, symbiont‐mediated acclimatization to stable, host‐regulated acclimatization that may influence environmental sensitivity and resistance. Representative time‐series icons illustrate the relative magnitude of seasonal variation in host and symbiont metrics observed for each genus.

Quantitative spatio‐temporal host and symbiont physiology is key to both a mechanistic understanding of coral biology and improving the diagnostics of climate change consequences for reef‐building corals. Our study of seasonal acclimatization in three dominant Indo‐Pacific reef‐building coral genera reveals contrasting physiological and symbiotic strategies that are consistent with previously described differences in environmental tolerance and ecological distribution among these genera (Figure 8). The more environmentally sensitive and symbiotic generalist taxa, *Acropora* and *Pocillopora*, follow a symbiont‐driven boom‐and‐bust cycle, referring to recurrent expansion and contraction of symbiont physiological performance (e.g., density, biomass, and photosynthetic activity), which was influenced primarily by changing light levels and, secondarily, by seasonal temperature fluctuations. In contrast, the environmentally resistant and symbiotic specialist, massive *Porites*, exhibits greater seasonal stability and greater host responsiveness to temperature, compared to light, across seasons (Figure 8). Although physiological differences between cryptic holobionts explained less variance than seasonal effects, these differences were significant. Cryptic lineages of *Pocillopora* and *Porites* exhibited distinct symbiont communities and thus physiological responses, highlighting the critical importance of identifying cryptic lineages and their symbiont communities to interpret physiology and performance in reef‐building corals. Our findings support the hypothesis that the environmental sensitivity of *Acropora* and *Pocillopora* may, in part, be associated with wide‐ranging symbiotic fluctuations contributing to strain on the symbiosis (e.g., competition and destabilization (Cunning and Baker 2013; McIlroy et al. 2020); excess reactive oxygen species (ROS)/reactive nitrogen species (RNS) production (Gibbin et al. 2014; Roth 2014); nutrient stress (Cui et al. 2022; Krueger et al. 2020; Rädecker et al. 2021)), which increase the sensitivity to, and negative outcomes of, dysbiosis. Further, our work highlights the need for characterizing seasonal baselines of coral species for trait‐based analysis (Edmunds and Putnam 2020; Madin et al. 2016), stress tolerance assays (Cunning et al. 2024), and mechanistic interpretation (Helgoe et al. 2024; Scheufen, Krämer, et al. 2017), all of which are key to our capacity to forecast reef futures.

### 4.1 Contrasting Seasonal Symbiotic Dynamics Among Coral Genera

Seasonal changes in this study were characterized by well‐documented biological drivers of light and temperature. Notably, these factors are among the strongest drivers of symbiotic photoacclimation (Fitt et al. 2000; Scheufen, Iglesias‐Prieto, and Enríquez 2017) and host biomass (Fitt et al. 2000; Thornhill et al. 2011), and their interactions have known synergistic and negative impacts for symbiosis (Venn et al. 2008). Light, for example, drives changes in aerial and per cell chlorophyll concentration (Scheufen, Iglesias‐Prieto, and Enríquez 2017) and thus photosynthetic rates, with implications for carbon translocation and symbiont and host biomass accumulation and thermal sensitivity (Scheufen, Krämer, et al. 2017). Temperature strongly influences enzymatic rates, and thus metabolic demand, contributing to summer biomass thinning and winter thickening of the host (Edmunds 2006, 2009; Fitt et al. 2000). In Moʻorea, the warm, high light season is characteristic of January and March time points; whereas September represents a cool, high light period; and November a cool, low light period. In light of this, it is expected that the host and symbiotic identity and host‐symbiont flexibility would transduce the light and temperature variability through time to emerge in contrasting acclimatization patterns across our timeseries.

The more environmentally sensitive taxa, *Acropora* and *Pocillopora*, are symbiotic generalists, hosting a wider diversity of symbiont taxa in comparison to the tolerant *Porites* corals, which consistently associated with specialized *Cladocopium* C15 lineages and exhibited more stable seasonal symbiont physiology. The predominance of *Durusdinium* in *Acropora pulchra* was consistent with previous reports from the same reef before the 2014–2017 global bleaching event (Meistertzheim et al. 2019) and earlier surveys documenting mixed associations with *Symbiodinium*, *Cladocopium*, and *Durusdinium* (Putnam et al. 2012; Rouzé et al. 2017). Across genera, seasonal increases in temperature and light resulted in photoacclimation in the holobiont in which symbiont productivity (i.e., density and photosynthesis rates) and metabolic demand (i.e., respiration) increased in the warmer months whereas cooler months were associated with increased cell‐specific chlorophyll and host biomass. Because symbiont taxa differ in photosynthetic performance (Brading et al. 2011, 2013) and carbon translocation (Allen‐Waller and Barott 2023; Stat et al. 2008), generating variable internal environments in the host (Al‐Horani 2005; Dellaert and Putnam 2023; Kühl et al. 1995), holobionts with distinct symbiont communities would be expected to respond differently to seasonal variability.

Consequently, seasonal shifts in symbiont density and photosynthesis across symbiont species and environmental contexts alter the host intracellular environment, requiring energetic investment to maintain homeostasis (Capasso et al. 2021; Tresguerres et al. 2017; Vidal‐Dupiol et al. 2013; Wall et al. 2020). Corals compartmentalize and maintain pH microenvironments that differ from the surrounding seawater, with conditions strongly influenced by symbiont photosynthesis (Barott et al. 2015; Gibbin et al. 2014; Laurent et al. 2013; Venn et al. 2009, 2025). Fluctuations in photosynthesis and symbiont densities can also generate rapid changes in tissue oxygen concentrations (Kühl et al. 1995; Wangpraseurt et al. 2012, 2016), potentially activating hypoxia response pathways (Alderdice et al. 2021; Glass and Barott 2025) and increasing needs for antioxidant defenses (Alderdice et al. 2022; Helgoe et al. 2024; Roth 2014; Weis 2008). In our study, *Acropora* demonstrated the greatest variability in photosynthetic rates across seasons (15%–26% between seasonal time points with maximum change in September), compared to 3%–10% change in *Pocillopora* (maximum change in November) and 3%–20% change in *Porites* (maximum change in September; Figure S7C). We posit that greater symbiont‐driven variability in *Acropora pulchra* may result in higher energetic costs to the host and contribute to increased environmental sensitivity.

*Acropora* and *Pocillopora* are often categorized as competitive and weedy species (Darling et al. 2012), with diverse and flexible symbiotic relationships (Putnam et al. 2012), while in contrast, massive *Porites* display higher stress tolerance (Darling et al. 2012) with more stable, high‐fidelity symbioses (Putnam et al. 2012). Our results identify divergent symbiotic seasonal acclimatization strategies in *Acropora, Pocillopora*, and *Porites*, representing a spectrum of generalist (high symbiotic seasonal acclimatization) to specialist (low symbiotic seasonal acclimatization) associations (i.e., higher vs. lower variance explained in symbiont physiology by time in Figure 6). Previously widely documented ecological data, particularly following bleaching events, indicate generalist and specialist symbiotic and life history features have been associated with differences in physiological stability and stress tolerance (Baird and Marshall 2002; Hughes et al. 2018; Loya et al. 2001; McClanahan et al. 2004; Pratchett et al. 2013; Putnam et al. 2012; Silverstein et al. 2012). While these seasonal “boom‐and‐bust” physiological dynamics may provide greater acclimatization plasticity, we posit that physiological flexibility can place strain on symbiotic nutritional relationships and therefore contribute to observed variation in holobiont ecological success (Loya et al. 2001). In particular, generalist relationships with high temporal variation in symbiont density and photophysiology (i.e., *Acropora*) may lead to increased symbiont competition, destabilization, and nutrient regulation imbalances that increase susceptibility to environmental stress, as compared to species with more stable symbiotic strategies (i.e., *Porites*).

While symbiotic flexibility may provide corals with the capacity to associate with symbiont taxa optimized for variable environmental conditions, there may be an increased risk of destabilization of symbiotic nutritional interactions. In *Acropora*, large seasonal swings in metabolic rates and energy reserves (i.e., 16%–17% change in host biomass, 9%–40% change in symbiont biomass, and 11%–39% change in respiration) may compromise host regulation of symbiont density (Cunning and Baker 2013), by disrupting the balance and availability of key nutrients, particularly nitrogen, which plays a central role in host‐mediated symbiont population control (Falkowski et al. 1993; Pogoreutz et al. 2017; Rädecker et al. 2015; Rädecker et al. 2023; Xiang et al. 2020). Therefore, in periods of highly fluctuating metabolism and physiological state as we documented across seasons, nutrient limitation and symbiont regulation may become less predictable, allowing symbiont communities to proliferate (Falkowski et al. 1993; McIlroy et al. 2022; Rädecker et al. 2015; Rädecker et al. 2023). Periods of symbiont overpopulation and destabilization are associated with increased bleaching risk (Cunning and Baker 2013; Wooldridge 2020), exacerbated by the production of excess ROS/RNS and triggering oxidative stress pathways that compromise symbiotic stability (Gibbin et al. 2014; Roth 2014). In our study, *Acropora* was the only taxa to exhibit symbiont growth during the seasonal warm period of March (14% increase), while *Pocillopora* (11% reduction) and *Porites* (5% reduction) showed slight decreases in density during this period, suggesting that the risk of symbiont overpopulation and destabilization is higher for *Acropora*.

Seasonal fluctuations in symbiont‐host dynamics have downstream effects for nutrient exchange and energy acquisition in the holobiont. Critically, uptake and translocation of carbon and nitrogen shift depend on symbiont density and host:symbiont ratios (McIlroy et al. 2022). Therefore, when symbiont densities surge, as seen in *Acropora* during peak growth periods in January–March in this study (i.e., 14% increase in March followed by a 24% decrease in September and 50% decrease in November relative to January), nutrient demand may outpace supply (Cui et al. 2022; Falkowski et al. 1993; Krueger et al. 2020; Xiang et al. 2020) and reduce energy availability in the host (Anthony et al. 2009; Cunning et al. 2017; Hoogenboom et al. 2010; Rädecker et al. 2018; Wooldridge 2013). Reductions in carbon sharing may, in particular, create energetic shortfalls in the host that may be detrimental if occurring during or before periods of high metabolic demand, such as reproduction and bleaching (Leinbach et al. 2021). In contrast to the variable seasonal changes seen in *Acropora, Porites* maintained relatively stable symbiont densities during seasonal warm periods (4%–6% reductions in March–September) and high‐fidelity associations with C15 symbionts, consistent with more stable nutrient exchange and energy availability across seasons. Indeed, we documented stable host biomass throughout seasonal cycles in *Porites* (5%–17% increases in biomass across all seasons) compared to losses in *Acropora* and *Pocillopora* (16% and 11%, respectively). Higher stability in nutrient and carbon exchange dynamics and biomass storage may contribute to the observed higher stress tolerance across studies in *Porites* (Baird and Marshall 2002; Hughes et al. 2018; Loya et al. 2001; McClanahan et al. 2004; Pratchett et al. 2013).

Our findings suggest that symbiont competition in generalist hosts, while allowing for potentially beneficial physiological flexibility, may introduce instability that compromises host tolerance under fluctuating or stressful conditions. Overall, our study suggests that both symbiotic strategy and host life history contribute to the stability of coral responses to environmental variation. Corals with fast growth and flexible, “boom‐and‐bust” dynamics, such as *Acropora*, which exhibit higher metabolic and symbiotic flexibility, may achieve short‐term gains but may also be prone to sharp declines, as documented during bleaching events (Baird and Marshall 2002; Hughes et al. 2018; Loya et al. 2001; McClanahan et al. 2004; Pratchett et al. 2013). In contrast, corals like *Porites*, which maintain slower growth and more stable, specialized symbiotic associations, appear to sustain more consistent energy supply across seasons. This stability may help buffer symbiotic stress under environmental extremes and avoid the energetic volatility inherent to boom‐and‐bust strategies. Thus, while flexibility can offer short‐term advantages, long‐term resilience may depend more on the steadiness of stable partnerships and conservative growth strategies.

### 4.2 Identifying Seasonal Baselines and Physiological Strategies Improves Interpretation of Ecological Outcomes

Our study highlights the importance of characterizing the seasonal physiological baseline of corals and their symbionts to understand and interpret performance across space and time. Therefore, seasonal taxa‐specific context is likely important for interpreting changes in performance and physiology. Because of this variation, we recommend that researchers provide seasonal physiological baselines to contextualize measurements of performance and stress tolerance. Previous studies have shown that thermal tolerance thresholds can shift seasonally, likely reflecting changes in metabolic state. For example, *P. verrucosa* corals in the Red Sea show up to a 3°C variation in thermal thresholds depending on the season, with *Acropora* corals showing a 1°C variation (García et al. 2024). Periods of reduced tolerance were seen in cooler seasonal periods and may be a result of shifts in metabolism between seasons (García et al. 2024). Further, *P. damicornis* winter bleaching thresholds were 1°C lower than summer thresholds (Berkelmans and Willis 1999). In contrast, juvenile *P. damicornis* corals reared in seasonal cool temperatures exhibited greater biomass reserves and higher thermal tolerance (Huffmyer et al. 2021). Our results underscore these prior findings and provide detailed physiological evidence that seasonal metabolic baselines provide important context for interpreting resilience and performance. Although seasonal acclimatization does not necessarily predict responses to extreme climate events, baseline physiological state provides important context for interpreting stress responses and understanding potential mechanisms underlying differences among coral taxa.

An important limitation of this study is that sampling was conducted at four seasonal time points rather than continuously throughout the year. However, these time points spanned a range of environmental conditions, including seasonal cool and warm periods (Figure 1A). As a result, we cannot determine the precise timing of physiological transitions between the seasonal states we observed or whether additional short‐term fluctuations occurred between warm and cool periods. Future studies with higher temporal resolution would help distinguish gradual seasonal acclimatization from short‐lived responses to episodic events, while the broad seasonal patterns identified here provide a baseline for understanding annual physiological strategies among coral holobionts. Further, some colonies went missing during this study due to tag loss, colony mortality/dislodgement, or inability to locate colonies; however, the fate of missing colonies could not be definitively classified by field observations and the recovery of some colonies after one or more missed sampling events demonstrates that missingness did not necessarily indicate colony mortality. Although missingness differed among genera, it was not associated with site or baseline physiological state within genera, and because analyses were conducted for each genus separately, colony missingness was unlikely to introduce systematic bias.

### 4.3 Identifying the Presence of Cryptic Lineages Is Critical for Interpreting Physiological Responses

Identifying cryptic coral species is important for interpreting physiological responses because each species hosts distinct symbiont communities and exhibits unique physiological traits. Cryptic species are common in *Pocillopora* (Burgess et al. 2021), and symbiont composition is tightly linked to host species identity (Johnston et al. 2022; Turnham et al. 2021). In our study, *Pocillopora tuahiniensis* hosted *Cladocopium pacificum*, whereas *P. meandrina* hosted *Cladocopium latusorum* (Johnston et al. 2022; Millán‐Márquez et al. 2024), and these communities did not vary by site. These species‐specific associations were accompanied by differences in symbiont traits including chlorophyll content and biomass, indicating that differences in symbiont communities can lead to distinct physiological responses across cryptic holobiont lineages. *Porites* showed high fidelity to *Cladocopium* C15 symbionts in our study, yet each lineage (i.e., *P. evermanni* and *P. lobata/lutea*) hosted distinct C15 subtypes, similar to observations in *Porites* corals in Palau (Grupstra et al. 2024) and Kiritimati (Starko et al. 2023). These differences were also seen in function with symbiont (e.g., symbiont density and photosynthetic traits) and host physiology (e.g., biomass and antioxidant capacity) varying with community composition. Indeed, other work in *Porites* demonstrated variation in photophysiology and thermal tolerance between host lineages, as well as variation in associated bacterial communities (Grupstra et al. 2024).

Collectively, our results underscore that physiological responses cannot be generalized across morphologically similar coral species within the same genus. Cryptic species differ in both their symbiotic relationships (Turnham et al. 2021) and their physiological performance (Burgess et al. 2021), making species‐level resolution important for understanding coral environmental resistance and resilience. In this study, the *Porites lobata/lutea* lineage was only found at two of our sites (Vaipahu and Orovau), while *Porites evermanni* was found at all three sites. Because physiological responses varied between *Porites* species, without species identification we would have falsely attributed variation in physiology between species to site effects. This has also been identified as a potential confounding factor in other studies (Starko et al. 2023). Cryptic species in *Pocillopora* and *Porites* corals are difficult to identify visually with morphology alone (Forsman et al. 2009; Grupstra et al. 2024; Johnston et al. 2022), and we recommend the inclusion of molecular identification of coral genera and associated symbionts to more accurately account for cryptic lineage effects.

## 5 Conclusions

In this study, we describe physiological strategies for seasonal acclimatization in three major reef‐building coral genera in Moʻorea, French Polynesia ranging from the stable, high‐fidelity strategy in *Porites* to the boom‐and‐bust pattern of *Acropora* and the muted but flexible response of *Pocillopora*. We hypothesize that these distinct physiological strategies may influence resilience to environmental stress and reef trajectories under changing conditions and highlight the importance of considering both host and symbiont contributions to holobiont function. We emphasize that characterizing seasonal baselines provides important context for understanding coral performance and informing predictions about responses to future stress. Together, our findings underscore that physiological and symbiotic strategies are likely to contribute to coral response to environmental influences and that these strategies may shape ecological distribution and success.

## Author Contributions

**Ariana S. Huffmyer:** data curation (lead), formal analysis (lead), visualization (lead), writing – original draft (lead), writing – review and editing (lead). **Jose M. Eirin‐Lopez:** conceptualization (supporting), funding acquisition (equal), investigation (supporting), writing – review and editing (supporting). **Steven B. Roberts:** conceptualization (supporting), funding acquisition (equal), writing – review and editing (supporting). **Francis J. Oliaro:** investigation (supporting). **Ross Cunning:** conceptualization (supporting), data curation (supporting), funding acquisition (equal), investigation (supporting), methodology (supporting), project administration (supporting), writing – review and editing (supporting). **Roger M. Nisbet:** conceptualization (supporting), funding acquisition (equal), writing – review and editing (supporting). **Hollie M. Putnam:** conceptualization (lead), data curation (equal), formal analysis (supporting), funding acquisition (equal), investigation (lead), methodology (lead), project administration (lead), supervision (lead), visualization (supporting), writing – original draft (supporting), writing – review and editing (supporting). **Zoe Dellaert:** investigation (supporting), writing – review and editing (supporting). **Kevin H. Wong:** investigation (supporting), writing – review and editing (supporting). **Danielle M. Becker:** investigation (supporting), writing – review and editing (supporting). **Emma L. Strand:** formal analysis (supporting), writing – original draft (supporting), writing – review and editing (supporting). **Serena Hackerott:** formal analysis (supporting), writing – original draft (supporting), writing – review and editing (supporting). **Ferdinand Pfab:** formal analysis (supporting), writing – review and editing (supporting). **Juliet M. Wong:** investigation (supporting), writing – review and editing (supporting). **Dennis Conetta:** investigation (supporting), writing – review and editing (supporting). **Kristina X. Terpis:** investigation (supporting).

## Funding

This work was supported by Division of Ocean Sciences, 2205966, 2224354. Gordon and Betty Moore Foundation. National Science Foundation Graduate Research Fellowship Program. Directorate for Biological Sciences, 1921149, 1921356, 1921402, 1921425, 1921465.

## Conflicts of Interest

The authors declare no conflicts of interest.

## Supporting information


**Table S1:** Sample sizes of the number of colonies sampled across time points for each genus, holobiont, and site. Holobiont indicates host haplotype and associated symbionts.
**Table S2:** Effect of genus on multivariate physiology. Permutational analysis of variance (PERMANOVA) and permutational analysis of dispersion (PERMDISP) analyses conducted for biological level (combined responses, host, symbiont) separately with genus as the main effect. Bold indicates *p* < 0.05. DF, degrees of freedom; SS, sum of squares. Tests run with 999 permutations.
**Table S3:** Pairwise multivariate analysis comparisons between holobionts within the *Pocillopora* and *Porites* genera. Permutational analysis of variance (PERMANOVA) and permutational analysis of dispersion (PERMDISP) analyses conducted for biological level (combined responses, host, symbiont) separately. Posthoc comparisons between holobionts within each genus conducted using pairwise PERMANOVA and Tukey HSD comparisons (PERMDISP tests). Bold indicates *p* < 0.05. DF, degrees of freedom; SS, sum of squares. Holobiont indicates host haplotype and associated symbionts.
**Table S4:** Permutational multivariate analysis of symbiont communities within each genus. PERMANOVA analyses conducted on relative abundance of ITS2 profiles for each genus separately. Holobiont, time point, site, and the interaction of time and site were included as main effects. Bold indicates *p* < 0.05. DF, degrees of freedom; SS, sum of squares. Holobiont refers to the host genetic haplotype and associated symbiont communities.
**Table S5:** Analysis of variance (3‐way ANOVA) tests for effects of site, ITS2 profile, and holobiont on relative abundance of Symbiodiniaceae taxa that comprise > 1% of total relative abundance. DF, degrees of freedom; SS, sum of squares. Bold indicates *p* < 0.05.
**Table S6:** Variance explained by main effects of multivariate physiology. Variance partitoning analyses conducted for each genus, biological level (combined responses, host, symbiont), and effects of site, time, and holobiont identity (i.e., coral host haplotype and associated symbiont community) for any term explaining > 1% of variance. Terms explaining < 1% of variance are not shown. The percent of the variance explained by each factor individually. *F* test statistics and *p*‐values for each main effect determined from ANOVA‐like permutation analyses of partial redundancy analyses for each individual main effect controlling for all other main effects.
**Table S7:** Univariate linear mixed effect model analysis of host responses. All responses were log+1 transformed. Linear mixed effect models were conducted for each genus and included timepoint, site, and their interactions as main effects with colony nested within holobiont (for *Porites* and *Pocillopora* only) as a random intercept. Significance determined with Type III analysis of variance tests. Den DF, denominator degrees of freedom; Num DF, numerator degrees of freedom; SS, sum of squares. Bold indicates *p* < 0.05. CRE indicates copper reducing elements. AFDW indicates ash‐free dry weight. Holobiont indicates host haplotype and associated symbionts.
**Table S8:** Univariate linear mixed effect model analysis of symbiont responses. All responses were log+1 transformed. Linear mixed effect models were run for each genus and included timepoint, site, and their interactions as main effects with colony nested within holobiont (for *Pocillopora* and *Porites* only) as a random intercept. Significance determined with Type III analysis of variance tests. Den DF, denominator degrees of freedom; Num DF, numerator degrees of freedom; SS, sum of squares. Bold indicates *p* < 0.05. PAR indicates photosynthetically active irradiance. AFDW indicates ash‐free dry weight. Holobiont indicates host haplotype and associated symbionts.
**Table S9:** Permutational multivariate analysis of variance of physiology. PERMANOVA analyses conducted for each species and biological level (combined responses, host, and symbiont) separately. Time point, site, and their interaction were included as main effects. Holobiont identity (i.e., host haplotype and associated symbiont communities) was also included in *Pocillopora* and *Porites* PERMANOVA models. Omega *R* ^2^ indicates *R* ^2^ corrected for degrees of freedom. Bold indicates *p* < 0.05. DF, degrees of freedom; SS, sum of squares. Tests run with 999 permutations.
**Table S10:** Permutational multivariate analysis of dispersion. PERMDISP analyses conducted for each species, biological level (combined responses, host, symbiont), and effects of site, time, and holobiont separately. Bold indicates *p* < 0.05. DF, degrees of freedom; SS, sum of squares. Holobiont refers to host haplotype and associated symbionts.
**Table S11:** Significance of variance explained by main effects on multivariate physiology. Variance partitoning analyses conducted for each genus, biological level (combined responses, host, symbiont), and effects of site, time, and holobiont identity (i.e., coral host haplotype and associated symbiont community). Significance of individual main effects tested by testing each main effect while controlling for the other main effects in turn. *F* test statistics and *p*‐values for each main effect determined from ANOVA‐like permutation analyses of partial redundancy analyses (RDA). DF, degrees of freedom. Holobiont main effect refers to the host genetic haplotype and associated symbiont communities. Combined responses are all host and symbiont responses analyzed together. Bold indicates *p* < 0.05.
**Table S12:** Evaluation of random effects in univariate linear mixed effect model analysis of host responses. ANOVA‐like analysis of random effects of linear mixed effect models on each response using single term deletions. AIC, Akaike Information Criterion; DF, degrees of freedom; LogLik, log likelihood; LRT, likelihood ratio test. All responses were log+1 transformed in linear mixed effect model analysis. Linear mixed effect models were conducted for each genus and included timepoint, site, and their interactions as main effects with colony nested within holobiont (for *Porites* and *Pocillopora* only) as random effects. Bold indicates *p* < 0.05. CRE indicates copper reducing elements. AFDW indicates ash‐free dry weight. Holobiont indicates host haplotype and associated symbionts.
**Table S13:** Evaluation of random effects in univariate linear mixed effect model analysis of symbiont responses. ANOVA‐like analysis of random effects of linear mixed effect models on each response using single term deletions. AIC, Akaike Information Criterion; DF, degrees of freedom; LogLik, log likelihood; LRT, likelihood ratio test. All responses were log+1 transformed in linear mixed effect model analysis. Linear mixed effect models were conducted for each genus and included timepoint, site, and their interactions as main effects with colony nested within holobiont (for *Porites* and *Pocillopora* only) as random effects. Bold indicates *p* < 0.05. PAR indicates photosynthetically active irradiance. AFDW indicates ash‐free dry weight. Holobiont indicates host haplotype and associated symbionts.
**Table S14:** Redundancy analysis of variance constrained in symbiont and host physiology by environmental characteristics. *p*‐values of each term from redundancy analysis (RDA) and model analysis of variance constrained in host and symbiont of each genus by host and symbiont responses with holobiont identity included in models for each biological level. Main effects were mean light (solar radiance in kWh m^−2^) and mean temperature (°C) that were scaled for analysis. Gray boxes indicate no significant effect. Bold indicates *p* < 0.05.
**Table S15:** Redundancy analysis of variance constrained in symbiont ITS2 communities by host and symbiont responses. Distance‐based RDA analysis of variance constrained in symbiont communities of each species by host and symbiont responses (Figure 2). Variance constrained by host and symbiont responses on symbiont communities in each species expresed as a percentage of variance in symbiont ITS2 community explained by significant models. Bold indicates *p* < 0.05. DF, degrees of freedom/number of responses tested at each biological level; SS, sum of squares.
**Table S16:** Redundancy analysis of variance constrained in symbiont ITS2 communities by host and symbiont responses at the genus level. *p*‐values of each term from distance‐based RDA (dbRDA) analysis of variance constrained in symbiont communities of each genus by host and symbiont responses with holobiont identity included in models for each biological level. Significance of variance explained in symbiont communities by each host and symbiont response as analyzed with dbRDA models. Gray boxes indicate no significant effect. Bold indicates *p* < 0.05.

**Figure S1:** Species trees for (A) *Acropora*, (B) *Porites*, and (C) *Pocillopora* (mtORF) and (D) RFLP gel for *Pocillopora* (PocHistone).
**Figure S2:** Principal components analysis of symbiont community. Symbiont communities visualized by holobiont identity for *Pocillopora* (left) and *Porites* (right) genera. In *Pocillopora*, gray indicates *Pocillopora meandrina* and black indicates *Pocillopora tuahiniensis*. In *Porites*, gray indicates *Porites evermanni* and black indicates *Porites lobata/lutea*. *p*‐values indicate significance of permutational analysis of variance (PERMANOVA) tests of the effect of holobiont on symbiont community composition (ITS2 type profile relative abundance).
**Figure S3:** Host physiological responses across sites and time points. Multivariate physiological trajectories in (A) *Acropora pulchra*, (B) *Pocillopora* spp., and (C) *Porites* spp. across site and time as visualized with principal components analyses. Trajectory arrows display the centroid of multivariate physiology of each species across time points, with the arrows beginning at the centroid of January 2020 samples and ending at the centroid of November 2020 samples. Color indicates site (Orovau = blue, Vaipahu = cyan, Matotia = orange). Biplots displayed for *Acropora pulchra* (D), *Pocillopora* spp. (E), and *Porites* spp. (F). Text indicates significance of site, time point, and the interaction using permutational analysis of variance tests. R_D_ indicates respiration.
**Figure S4:** Symbiont physiological responses across sites and time points. Multivariate physiological trajectories in (A) *Acropora pulchra*, (B) *Pocillopora* spp., and (C) *Porites* spp. across site and time as visualized with principal components analyses. Trajectory arrows display the centroid of multivariate physiology of each species across time points, with the arrows beginning at the centroid of January 2020 samples and ending at the centroid of November 2020 samples. Color indicates site (Orovau = blue, Vaipahu = cyan, Matotia = orange). Biplots displayed for *Acropora pulchra* (D), *Pocillopora* spp. (E), and *Porites* spp. (F) Text indicates significance of site, time point, and the interaction using permutational analysis of variance tests. P_MAX_ indicates maximal photosynthesis; I_C_ indicates compensation irradiance; I_K_ indicates saturating irradiance.
**Figure S5:** Host responses across site and time point for each genus. Mean ± standard error of mean of host responses across time point (x‐axis) and site (blue = Orovau, cyan = Vaipahu, orange = Matotia). Plots are faceted by genus. Host responses include (A) antioxidant capacity (μmol copper reducing elements mg AFDW^−1^), (B) host biomass (mg AFDW cm^−2^), (C) host protein (mg protein mg AFDW^−1^), (D) respiration (R_D_; μmol O_2_ cm^−2^ h^−1^), (E) calcification (μmol CaCO_3_ cm^−2^ h^−1^). Time points are ordered as January, March, September, and November 2020.
**Figure S6:** Symbiont responses across site and time point for each genus. Mean ± standard error of mean of symbiont responses across time point (x‐axis) and site (blue = Orovau, cyan = Vaipahu, orange = Matotia). Plots are faceted by genus. Responses include (A) symbiont cell density (cells mg AFDW^−1^), (B) symbiont biomass (mg AFDW cm^−2^), (C) maximal photosynthesis (P_MAX_; μmol O_2_ cm^−2^ h^−1^), (D) apparent quantum yield (AQY; expressed as a proportion), (E) saturating irradiance (I_K_; PAR), (F) compensation irradiance (I_C_; PAR), (G) total chlorophyll (chl *a* + chl *c*_*2*_) (μg pigment mg AFDW^−1^), (H) total cell‐specific chlorophyll (chl *a* + chl *c*_*2*_) (μg pigment cell^−1^), (I) S:H biomass. Time points are ordered as January, March, September, and November 2020.
**Figure S7:** Physiological responses across time points for each *Pocillopora* holobiont—*P. meandrina* (black) and *P. tuahiniensis* (gray) in host (left) and symbiont (right) responses. Trajectory arrows display the centroid of multivariate physiology of each holobiont across time points, with the arrows beginning at the centroid of January 2020 and ending at the centroid of November 2020. *p*‐values indicate significance of permutational analysis of variance (PERMANOVA) and permutational analysis of dispersion (PERMDISP) tests.
**Figure S8:** Host responses across site and time point for each haplotype in the *Pocillopora* genus. Mean ± standard error of mean of host responses across time point (x‐axis) and haplotypes in *Pocillopora* (black = *P. meandrina*, gray = *P. tuahiniensis*). Host responses include (A) antioxidant capacity (μmol CRE mg AFDW^−1^), (B) host biomass (mg AFDW cm^−2^), (C) host protein (mg protein AFDW^−1^), (D) respiration (R_D_; μmol O_2_ cm^−2^ h^−1^), (E) calcification (μmol CaCO_3_ cm^−2^ h^−1^). Time points are ordered as January, March, September, and November 2020.
**Figure S9:** Symbiont responses across site and time point for each holobiont in the *Pocillopora* genus. Mean ± standard error of mean of symbiont responses across time point (x‐axis) and haplotypes in *Pocillopora* (black = *P. meandrina*, gray = *P. tuahiniensis*). Host responses include (A) symbiont cell density (cells mg AFDW^−1^), (B) symbiont biomass (mg AFDW cm^−2^), (C) maximal photosynthesis (P_MAX_; μmol O_2_ cm^−2^ h^−1^), (D) apparent quantum yield (AQY; expressed as a proportion), (E) saturating irradiance (I_K_; PAR), (F) compensation irradiance (I_C_; PAR), (G) total chlorophyll (chl *a* + chl *c*_*2*_) (μg pigment mg AFDW^−1^), (H) total cell‐specific chlorophyll (chl *a* + chl *c*_*2*_; μg pigment cell^−1^), (I) S:H biomass. Time points are ordered as January, March, September, and November 2020.
**Figure S10:** Physiological responses across time points for each *Porites* holobiont—*P. evermanni* (black) and *P. lobata/lutea* (gray) for host (left) and symbiont (right) responses. Trajectory arrows display the centroid of multivariate physiology of each holobiont across time points, with the arrows beginning at the centroid of January 2020 and ending at the centroid of November 2020. *p*‐values indicate significance of permutational analysis of variance (PERMANOVA) and permutational analysis of dispersion (PERMDISP) tests.
**Figure S11:** Host responses across site and time point for each holobiont in the *Porites* genus. Mean ± standard error of mean of host responses across time point (x‐axis) and haplotypes in *Porites* (black = *P. evermanni*, gray = *P. lobata/lutea*). Host responses include (A) antioxidant capacity (μmol CRE mg AFDW^−1^), (B) host biomass (mg AFDW cm^−2^), (C) host protein (mg protein AFDW^−1^), (D) respiration (R_D_; μmol O_2_ cm^−2^ h^−1^), (E) calcification (μmol CaCO_3_ cm^−2^ h^−1^). Time points are ordered as January, March, September, and November 2020.
**Figure S12:** Symbiont responses across site and time point for each holobiont in the *Porites* genus. Mean ± standard error of mean of symbiont responses across time point (x‐axis) and haplotypes in *Porites* (black = *P. evermanni*, gray = *P. lobata/lutea*). Host responses include (A) symbiont cell density (cells mg AFDW^−1^), (B) symbiont biomass (mg AFDW cm^−2^), (C) maximal photosynthesis (P_MAX_; μmol O_2_ cm^−2^ h^−1^), (D) apparent quantum yield (AQY; expressed as a proportion), (E) saturating irradiance (I_K_; PAR), (F) compensation irradiance (I_C_; PAR), (G) total chlorophyll (chl *a* + chl *c*_*2*_) (μg pigment mg AFDW^−1^), (H) total cell‐specific chlorophyll (chl *a* + chl *c*_*2*_; μg pigment cell^−1^), (I) S:H biomass. Time points are ordered as January, March, September, and November 2020.

## Data Availability

All data and code are openly available on GitHub (https://github.com/urol‐e5/timeseries), as a static release (https://github.com/urol‐e5/timeseries/releases/tag/v3.0), and on Zenodo (https://doi.org/10.5281/zenodo.21363719).
