# Supplementary material for: Seasonal Physiological Strategies Reveal Contrasting Host–Symbiont Dynamics Among Dominant Indo‐Pacific Reef‐Building Corals
Source: Ecol Evol. 2026 Jul 19;16(7):e74044. doi: 10.1002/ece3.74044 (PMC13381095; doi:10.1002/ece3.74044)
Supplement: Supplementary file 1 — Table S1: Sample sizes of the number of colonies sampled across time points for each genus, holobiont, and site. Holobiont indicates host haplotype and associated symbionts. Table S2: Effect of genus on multivariate physiology. Permutational analysis of variance (PERMANOVA) and permutational analysis of dispersion (PERMDISP) analyses conducted for biological level (combined responses, host, symbiont) separately with genus as the main effect. Bold indicates p < 0.05. DF, degrees of freedom; SS, sum of squares. Tests run with 999 permutations. Table S3: Pairwise multivariate analysis comparisons between holobionts within the Pocillopora and Porites genera. Permutational analysis of variance (PERMANOVA) and permutational analysis of dispersion (PERMDISP) analyses conducted for biological level (combined responses, host, symbiont) separately. Posthoc comparisons between holobionts within each genus conducted using pairwise PERMANOVA and Tukey HSD comparisons (PERMDISP tests). Bold indicates p < 0.05. DF, degrees of freedom; SS, sum of squares. Holobiont indicates host haplotype and associated symbionts. Table S4: Permutational multivariate analysis of symbiont communities within each genus. PERMANOVA analyses conducted on relative abundance of ITS2 profiles for each genus separately. Holobiont, time point, site, and the interaction of time and site were included as main effects. Bold indicates p < 0.05. DF, degrees of freedom; SS, sum of squares. Holobiont refers to the host genetic haplotype and associated symbiont communities. Table S5: Analysis of variance (3‐way ANOVA) tests for effects of site, ITS2 profile, and holobiont on relative abundance of Symbiodiniaceae taxa that comprise > 1% of total relative abundance. DF, degrees of freedom; SS, sum of squares. Bold indicates p < 0.05. Table S6: Variance explained by main effects of multivariate physiology. Variance partitoning analyses conducted for each genus, biological level (combined responses, host, symbiont), an [file ECE3-16-e74044-s002.pdf]

Table S1. Sample sizes of the number of colonies sampled across time points for each genus, holobiont, and site. Holobiont indicates host haplotype and associated symbionts.

Genus	Holobiont	Site	Number of colonies measured per time point			
			January	March	September	November
Acropora	*Acropora pulchra*	Vaipahu	13	9	2	6
		Orovau	14	6	10	12
		Matotia	15	9	9	11
Pocillopora	*Pocillopora meandrina*	Vaipahu	3	3	3	3
		Orovau	8	9	7	7
		Matotia	3	3	2	2
Pocillopora	*Pocillopora tuahinensis*	Vaipahu	12	11	10	12
		Orovau	5	6	6	5
		Matotia	12	12	10	11
Porites	*Porites evermanni*	Vaipahu	12	11	12	11
		Orovau	6	6	4	4
		Matotia	15	15	13	12
Porites	*Porites lobata lutea*	Vaipahu	3	3	3	3
		Orovau	9	9	8	8
		Matotia	0	0	0	0

Table S2. Effect of genus on multivariate physiology. Permutational analysis of variance (PERMANOVA) and permutational analysis of dispersion (PERMDISP) analyses conducted for biological level (combined responses, host, symbiont) separately with genus as the main effect. Bold indicates P<0.05. DF = degrees of freedom; SS = sum of squares. Tests run with 999 permutations.

Test	Biological Level	Main Effect	DF	SS	R2	F value	P-value
PERMANOVA	Combined	genus	2	1872.90	0.36	104.00	**0.001**
	Host	genus	2	835.20	0.43	145.30	**0.001**
	Symbiont	genus	2	1174.50	0.32	95.20	**0.001**
PERMDISP	Combined	genus	2	1.03E+12		70.87	**<0.001**
	Host	genus	2	164.28		98.49	**<0.001**
	Symbiont	genus	2	1.21E+12		82.91	**<0.001**

Table S3. Pairwise multivariate analysis comparisons between holobionts within the *Pocillopora* and *Porites* genera. Permutational analysis of variance **(**PERMANOVA) and permutational analysis of dispersion (PERMDISP) analyses conducted for biological level (combined responses, host, symbiont) separately. Posthoc comparisons between holobionts within each genus conducted using pairwise PERMANOVA and Tukey HSD comparisons (PERMDISP tests). Bold indicates P<0.05. DF = degrees of freedom; SS = sum of squares. Holobiont indicates host haplotype and associated symbionts.

Genus	Comparison	Biological Level	Pairwise PERMANOVA				Pairwise PERMDISP	
			DF	SS	F	Adjusted P-value	Difference	Adjusted P-value
Pocillopora	*P. tuahiniensis - P. meandrina*	Combined	1	1.25E+12	0.362	0.551	1272.123	0.999
		Host	1	1.543	3.136	0.690	0.077	0.989
		Symbiont	1	3.15E+06	8.46E-04	1.000	4184.311	0.999
Porites	*P. lobata/lutea - P. evermanni*	Combined	1	3.24E+11	49.733	**0.001**	-40077.049	0.102
		Host	1	18.037	3.357	0.590	0.396	0.163
		Symbiont	1	3.732	6.05E+01	**0.010**	-35248.049	0.148

Table S4. Permutational multivariate analysis of symbiont communities within each genus. PERMANOVA analyses conducted on relative abundance of ITS2 profiles for each genus separately. Holobiont, time point, site, and the interaction of time and site were included as main effects. Bold indicates P<0.05. DF = degrees of freedom; SS = sum of squares. Holobiont refers to the host genetic haplotype and associated symbiont communities.

Genus	Main Effect	DF	SS	R2	F value	P-value
Acropora	site	2	0.19	0.01	0.44	0.77
	timepoint	3	0.30	0.01	0.47	0.853
	site:timepoint	6	0.38	0.02	0.30	0.986
	residuals	107	22.64	0.96		
Pocillopora	holobiont	1	14.28	0.12	20.31	**0.002**
	timepoint	2	2.88	0.02	2.05	0.052
	site:timepoint	3	0.98	0.01	0.46	0.955
	holobiont	6	2.70	0.02	0.64	0.913
	residuals	145	102.00	0.83		
Porites	holobiont	1	31.06	0.29	63.04	**0.001**
	site	2	1.99	0.02	2.02	0.060
	timepoint	3	0.60	0.01	0.40	0.986
	site:timepoint	6	1.10	0.01	0.37	0.998
	residuals	149	73.408	0.68		

Table S5. Analysis of variance (3-way ANOVA) tests for effects of site, ITS2 profile, and holobiont on relative abundance of Symbiodiniaceae taxa that comprise >1% of total relative abundance. DF = degrees of freedom; SS = sum of squares. Bold indicates P<0.05.

Genus	Main Effect	DF	SS	F	P-value
Acropora	site	2	0.00	0.00	1.000
	profile	4	24.29	147.11	**<0.001**
	site:profile	6	0.08	0.24	0.961
Pocillopora	site	2	0.00	0.00	1.000
	profile	13	7.06	15.18	**<0.001**
	holobiont	1	0.00	0.00	1.000
	site:profile	26	1.66	1.78	**0.011**
	site:holobiont	2	0.00	0.00	1.000
	profile:holobiont	13	3.31	7.12	**<0.001**
	site:profile:holobiont	26	0.64	0.69	0.876
Porites	site	2	0.00	0.00	1.000
	profile	20	12.78	37.42	**<0.001**
	holobiont	1	0.00	0.00	1.000
	site:profile	40	3.24	4.74	**<0.001**
	site:holobiont	1	0.00	0.00	1.000
	profile:holobiont	20	5.83	17.07	**<0.001**
	site:profile:holobiont	20	0.14	0.42	0.988

Table S6. Variance explained by main effects of multivariate physiology. Variance partitoning analyses conducted for each genus, biological level (combined responses, host, symbiont), and effects of site, time, and holobiont identity (i.e. coral host haplotype and associated symbiont community) for any term explaining >1% of variance. Terms explaining <1% of variance are not shown. The percent of the variance explained by each factor individually. F test statistics and P-values for each main effect determined from ANOVA-like permutation analyses of partial redundancy analyses for each individual main effect controlling for all other main effects.

Genus	Biological Level	Effect	Variance Explained (%)
Acropora	Combined	timepoint	38.4
		site	3.1
		residuals	59.6
	Host	timepoint	26.7
		site	8.0
		residuals	66.7
	Symbiont	timepoint	39.2
		site	2.5
		residuals	59.4
Pocillopora	Combined	timepoint	28.4
		site	1.3
		residuals	70.4
	Host	timepoint	18.3
		site	3.1
		site:holobiont	1.3
		residuals	77.5
	Symbiont	timepoint	28.0
		residuals	71.6
	Combined	timepoint	19.3
		site	3.9
		holobiont	5.6
		site:holobiont	7.0
		residuals	63.8

Porites	Host	timepoint	28.0
		site	1.7
		site:holobiont	3.1
		residuals	67.7
	Symbiont	timepoint	18.1
		site	4.6
		holobiont	6.0
		site:holobiont	8.0
		residuals	62.0

Table S7. Univariate linear mixed effect model analysis of host responses. All responses were log+1 transformed. Linear mixed effect models were conducted for each genus and included timepoint, site, and their interactions as main effects with colony nested within holobiont (for *Porites* and *Pocillopora* only) as a random intercept. Significance determined with Type III analysis of variance tests. SS = sum of squares; Num DF = numerator degrees of freedom; Den DF = denominator degrees of freedom. Bold indicates P<0.05. CRE indicates copper reducing elements. AFDW indicates ash-free dry weight. Holobiont indicates host haplotype and associated symbionts.

Response	Genus	Main Effect	SS	Num DF	Den DF	F value	P-value
**HOST RESPONSES**							
Antioxidant Capacity (µmol CRE mg AFDW-1)	*Acropora*	timepoint	0.092	3	79.09	34.72	**<0.001**
		site	0.004	2	58.35	2.81	0.068
		timepoint:site	0.014	6	78.21	2.72	**0.019**
	Pocillopora	timepoint	0.043	3	123.00	33.43	**<0.001**
		site	0.001	2	123.00	1.06	0.349
		timepoint:site	0.005	6	123.00	2.00	0.071
	Porites	timepoint	0.119	3	94.65	21.51	**<0.001**
		site	0.013	2	43.25	3.55	**0.037**
		timepoint:site	0.019	6	94.59	1.74	0.119
Host Soluble Protein (mg protein mg AFDW-1)	*Acropora*	timepoint	0.231	3	75.41	27.23	**<0.001**
		site	0.038	2	46.22	6.66	**0.003**
		timepoint:site	0.011	6	74.31	0.66	0.680
	Pocillopora	timepoint	0.111	3	121.23	30.04	**<0.001**
		site	0.012	2	114.96	4.69	**0.011**
		timepoint:site	0.015	6	121.17	2.09	0.059
	Porites	timepoint	0.180	3	97.76	17.81	**<0.001**
		site	0.024	2	24.98	3.49	**0.039**
		timepoint:site	0.077	6	97.66	3.79	**0.002**
Host Biomass (mg AFDW cm-2)	*Acropora*	timepoint	0.323	3	77.49	3.14	**0.030**
		site	0.254	2	44.50	3.70	**0.033**
		timepoint:site	0.165	6	76.00	0.80	0.573
	Pocillopora	timepoint	1.195	3	87.24	19.44	**<0.001**
		site	0.233	2	34.76	5.68	**0.007**
		timepoint:site	0.512	6	86.86	4.17	**0.001**
	Porites	timepoint	0.635	3	102.48	3.03	**0.033**
		site	0.701	2	41.66	41.66	**0.011**
		timepoint:site	1.036	6	102.27	2.47	**0.028**

Respiration (RD) (µmol O2 cm-1 h-1)	*Acropora*	timepoint	0.368	3	69.52	13.91	**<0.001**
		site	0.134	2	31.74	7.57	**0.002**
		timepoint:site	0.067	6	67.42	1.26	0.286
	Pocillopora	timepoint	0.015	3	91.24	1.29	0.281
		site	0.045	2	39.58	5.77	**0.006**
		timepoint:site	0.113	6	90.90	4.86	**<0.001**
	Porites	timepoint	0.553	3	128.00	10.89	**<0.001**
		site	0.114	2	128.00	3.36	**0.038**
		timepoint:site	0.092	6	128.00	0.91	0.490
Calcification (µmol CaCO3 mg cm-2 h-1)	*Acropora*	timepoint	0.808	3	78.38	36.08	**<0.001**
		site	0.034	2	50.04	2.29	0.111
		timepoint:site	0.187	6	77.14	4.17	**0.001**
	Pocillopora	timepoint	0.429	3	101.71	11.46	**<0.001**
		site	0.014	2	45.61	0.56	0.575
		timepoint:site	0.152	6	101.33	2.03	0.068
	Porites	timepoint	7.236	3	128.00	63.45	**<0.001**
		site	0.266	2	128.00	3.51	**0.033**
		timepoint:site	0.655	6	128.00	2.87	**0.012**

Table S8. Univariate linear mixed effect model analysis of symbiont responses. All responses were log+1 transformed. Linear mixed effect models were run for each genus and included timepoint, site, and their interactions as main effects with colony nested within holobiont (for *Pocillopora* and *Porites* only) as a random intercept. Significance determined with Type III analysis of variance tests. SS = sum of squares; Num DF = numerator degrees of freedom; Den DF = denominator degrees of freedom. Bold indicates P<0.05. PAR indicates photosynthetically active irradiance. AFDW indicates ash-free dry weight. Holobiont indicates host haplotype and associated symbionts.

Response	Genus	Main Effect	SS	Num DF	Den DF	F value	P-value
**SYMBIONT RESPONSES**							
Symbiont Cell Density (cells mg AFDW-1)	*Acropora*	timepoint	23.200	3	92.00	64.26	**<0.001**
		site	0.173	2	92.00	0.72	0.489
		timepoint:site	2.154	6	92.00	2.98	**0.010**
	Pocillopora	timepoint	17.250	3	86.31	47.16	**<0.001**
		site	0.549	2	37.43	2.17	0.128
		timepoint:site	3.214	6	86.01	4.39	**0.001**
	Porites	timepoint	10.716	3	88.10	37.05	**<0.001**
		site	1.614	2	26.18	8.37	**0.002**
		timepoint:site	1.817	6	87.97	3.14	**0.008**
Symbiont Biomass (mg AFDW cm-2)	*Acropora*	timepoint	0.230	3	83.07	4.19	**0.008**
		site	0.156	2	49.10	4.25	**0.020**
		timepoint:site	0.247	6	81.77	2.25	**0.047**
	Pocillopora	timepoint	0.723	3	91.24	17.43	**<0.001**
		site	0.104	2	37.54	3.74	**0.033**
		timepoint:site	0.157	6	90.93	1.89	0.092
	Porites	timepoint	0.809	3	97.52	2.42	0.071
		site	0.596	2	37.74	2.67	0.082
		timepoint:site	0.604	6	97.26	0.90	0.495
Total Chlorophyll (chl a + chl c2) (µg chl mg AFDW-1)	*Acropora*	timepoint	3.481	3	92.00	11.52	**<0.001**
		site	0.694	2	92.00	3.44	**0.036**
		timepoint:site	1.926	6	92.00	3.19	**0.007**
	Pocillopora	timepoint	2.901	3	122.00	18.82	**<0.001**
		site	0.878	2	122.00	8.54	**<0.001**
		timepoint:site	1.660	6	122.00	5.39	**<0.001**
	Porites	timepoint	2.337	3	128.00	10.30	**<0.001**
		site	1.699	2	128.00	11.23	**<0.001**
		timepoint:site	1.045	6	128.00	2.30	**0.038**

Total Cell-Specific Chlorophyll (chl a + chl c2) (µg cell-1)	*Acropora*	timepoint	5.37E-10	3	7.13	13.12	**0.003**
		site	6.42E-11	2	6.18	2.35	0.174
		timepoint:site	7.13E-11	6	7.13	0.87	0.558
	Pocillopora	timepoint	1.04E-09	3	14.22	5.52	**0.010**
		site	3.78E-10	2	28.70	2.99	0.066
		timepoint:site	6.77E-10	6	14.19	1.79	0.173
	Porites	timepoint	4.12E-10	3	11.01	5.28	**0.017**
		site	1.23E-10	2	10.95	2.37	0.140
		timepoint:site	2.89E-10	6	11.01	1.85	0.179
Symbiont : Host Biomass	*Acropora*	timepoint	0.074	3	8.78	8.78	**<0.001**
		site	0.020	2	3.62	3.62	**0.034**
		timepoint:site	0.039	6	2.30	2.30	**0.043**
	Pocillopora	timepoint	0.162	3	121.02	30.46	**<0.001**
		site	0.007	2	121.30	1.98	0.143
		timepoint:site	0.029	6	121.02	2.73	**0.016**
	Porites	timepoint	0.039	3	95.67	5.57	**0.001**
		site	0.003	2	42.14	0.61	0.550
		timepoint:site	0.007	6	95.58	0.49	0.815
Maximal Photosynthesis (PMAX) (µmol O2 cm-1 h-1)	*Acropora*	timepoint	0.468	3	54.40	12.19	**<0.001**
		site	0.078	2	36.82	3.03	0.060
		timepoint:site	0.172	6	53.36	2.25	0.053
	Pocillopora	timepoint	0.211	3	95.86	7.29	**<0.001**
		site	0.059	2	28.21	3.04	0.064
		timepoint:site	0.054	6	95.49	0.93	0.479
	Porites	timepoint	0.582	3	128.00	4.93	**0.003**
		site	0.309	2	128.00	3.94	**0.022**
		timepoint:site	0.216	6	128.00	0.92	0.486
Apparent Quantum Yield (AQY)	*Acropora*	timepoint	3.57E-06	3	44.82	2.79	0.051
		site	1.21E-06	2	39.70	1.42	0.255
		timepoint:site	3.14E-06	6	44.55	1.22	0.312
	Pocillopora	timepoint	2.64E-05	3	92.35	11.47	**<0.001**
		site	4.66E-06	2	34.09	3.04	0.061
		timepoint:site	6.13E-06	6	91.88	1.33	0.250
	Porites	timepoint	1.53E-04	3	127.07	6.36	**<0.001**
		site	4.08E-05	2	103.25	2.54	0.840
		timepoint:site	6.15E-05	6	127.04	1.28	0.272
	Acropora	timepoint	1.469	3	59.57	7.34	**<0.001**
		site	0.117	2	44.49	0.88	0.423

		timepoint:site	0.570	6	58.71	1.42	0.221
Saturating Irradiance (IK) (PAR)	*Pocillopora*	timepoint	7.568	3	88.58	24.84	**<0.001**
		site	0.237	2	28.78	1.17	0.325
		timepoint:site	0.543	6	88.04	0.89	0.505
	Porites	timepoint	5.478	3	127.08	16.34	**<0.001**
		site	1.717	2	101.78	7.68	**<0.001**
		timepoint:site	2.164	6	127.04	3.23	**0.006**
Compensation Irradiance (IC) (PAR)	*Acropora*	timepoint	7.013	3	70.81	12.66	**<0.001**
		site	2.440	2	28.10	6.61	**0.004**
		timepoint:site	2.388	6	68.35	2.16	0.058
	Pocillopora	timepoint	2.659	3	94.09	4.91	**0.003**
		site	0.194	2	25.57	0.54	0.592
		timepoint:site	4.822	6	93.54	4.46	**<0.001**
	Porites	timepoint	3.378	3	91.92	8.72	**<0.001**
		site	0.669	2	28.52	2.59	0.093
		timepoint:site	2.930	6	91.71	3.78	**0.002**

Table S9. Permutational multivariate analysis of variance of physiology. PERMANOVA analyses conducted for each species and biological level (combined responses, host, and symbiont) separately. Time point, site, and their interaction were included as main effects. Holobiont identity (i.e., host haplotype and associated symbiont communities) was also included in *Pocillopora* and *Porites* PERMANOVA models. Omega R2 indicates R2 corrected for degrees of freedom. Bold indicates P<0.05. DF = degrees of freedom; SS = sum of squares. Tests run with 999 permutations.

Genus	Biological Level	Main Effect	DF	SS	R2	Omega R2	F value	P-value
Acropora	Combined	timepoint	3	423.98	0.30	0.30	15.67	**0.001**
		site	2	95.01	0.07	0.08	5.27	**0.001**
		timepoint:site	6	83.2	0.06	0.03	1.54	**0.025**
		residuals	90	811.91	0.57			
	Host	timepoint	3	196.82	0.38	0.39	23.65	**0.001**
		site	2	35.68	0.07	0.09	6.43	**0.001**
		timepoint:site	6	27.3	0.05	0.04	1.64	**0.033**
		residuals	92	255.21	0.50			
	Symbiont	timepoint	3	255.03	0.26	0.25	13.68	**0.001**
		site	2	60.94	0.06	0.07	4.90	**0.001**
		timepoint:site	6	61.66	0.06	0.03	1.65	**0.021**
		residuals	100	621.37	0.62			
Pocillopora	Combined	holobiont	1	67.75	0.04	0.16	7.19	**0.001**
		timepoint	3	442.85	0.24	0.19	15.67	**0.001**
		site	2	69.74	0.04	0.04	3.70	**0.001**
		timepoint:site	6	137.41	0.07	0.04	2.43	**0.001**
		residuals	120	1130.25	0.61			
	Host	holobiont	1	12.43	0.02	0.02	3.63	**0.005**
		timepoint	3	173.34	0.24	0.25	16.88	**0.001**
		site	2	22.18	0.03	0.03	3.24	**0.002**
		timepoint:site	6	61.73	0.09	0.08	3.01	**0.001**
		residuals	133	455.33	0.63			
	Symbiont	holobiont	1	45.11	0.04	0.04	7.15	**0.001**
		timepoint	3	282.66	0.22	0.22	14.94	**0.001**
		site	2	39.87	0.03	0.03	3.16	**0.003**
		timepoint:site	6	98.76	0.08	0.06	2.61	**0.001**
		residuals	133	838.62	0.64			

Porites	Combined	holobiont	1	258.36	0.13	0.04	28.05	**0.001**
		timepoint	3	329.09	0.17	0.25	11.91	**0.001**
		site	2	75.05	0.04	0.04	4.08	**0.001**
		timepoint:site	6	108.94	0.06	0.06	1.97	**0.001**
		residuals	126	1160.55	0.60			
	Host	holobiont	1	92.46	0.13	0.17	29.55	**0.001**
		timepoint	3	150.69	0.22	0.24	16.05	**0.001**
		site	2	22.42	0.03	0.04	3.58	**0.002**
		timepoint:site	6	33.90	0.05	0.03	1.81	**0.012**
		residuals	128	400.53	0.57			
	Symbiont	holobiont	1	186.77	0.13	0.16	30.78	**0.001**
		timepoint	3	202.11	0.14	0.16	11.1	**0.001**
		site	2	64.47	0.05	0.05	5.31	**0.001**
		timepoint:site	6	76.75	0.05	0.04	2.11	**0.001**
		residuals	144	873.9	0.62			

Table S10. Permutational multivariate analysis of dispersion. PERMDISP analyses conducted for each species, biological level (combined responses, host, symbiont), and effects of site, time, and holobiont separately. Bold indicates P<0.05. DF = degrees of freedom; SS = sum of squares. Holobiont refers to host haplotype and associated symbionts.

Genus	Biological Level	Effect	DF	SS	F value	P-value
Acropora	Combined	site	2	1.68	5.99	**0.004**
		timepoint	3	0.83	2.69	0.051
	Host	site	2	0.16	4.41	**0.015**
		timepoint	3	0.02	0.34	0.794
	Symbiont	site	2	2.07	6.56	**0.002**
		timepoint	3	1.34	3.76	**0.013**
Pocillopora	Combined	site	2	0.53	2.46	0.089
		holobiont	1	0.06	0.52	0.471
		timepoint	3	2.09	7.47	**<0.001**
	Host	site	2	0.08	1.70	0.186
		holobiont	1	0.02	0.75	0.389
		timepoint	3	0.15	2.81	**0.042**
	Symbiont	site	2	0.77	3.49	**0.033**
		holobiont	1	0.06	0.50	0.481
		timepoint	3	1.90	6.41	**<0.001**
Porites	Combined	site	2	2.35	8.87	**<0.001**
		holobiont	1	0.34	2.53	0.114
		timepoint	3	1.01	2.34	0.076
	Host	site	2	0.53	2.46	0.089
		holobiont	1	0.15	4.53	**0.035**
		timepoint	3	0.027	2.68	0.050
	Symbiont	site	2	2.43	8.73	**<0.001**
		holobiont	1	0.84	2.15	0.097
		timepoint	3	0.25	1.86	0.175

Table S11. Significance of variance explained by main effects on multivariate physiology. Variance partitoning analyses conducted for each genus, biological level (combined responses, host, symbiont), and effects of site, time, and holobiont identity (i.e. coral host haplotype and associated symbiont community). Significance of individual main effects tested by testing each main effect while controlling for the other main effects in turn. F test statistics and P-values for each main effect determined from ANOVA-like permutation analyses of partial redundancy analyses (RDA). DF = degrees of freedom. Holobiont main effect refers to the host genetic haplotype and associated symbiont communities. Combined responses are all host and symbiont responses analyzed together. Bold indicates P<0.05.

Genus	Biological Level	Effect Tested	Effect	DF	Variance	F	P-value
Acropora	Combined	time	site	3	0.40	22.23	**0.001**
		site	time	2	0.04	3.58	**0.001**
	Host	time	site	3	0.02	14.50	**0.001**
		site	time	2	0.01	7.01	**0.001**
	Symbiont	time	site	3	0.39	25.01	**0.001**
		site	time	2	0.03	3.24	**0.004**
Pocillopora	Combined	time	site	3	0.27	18.39	**0.001**
		time	holobiont	3	0.27	18.12	**0.001**
		site	time	2	0.02	2.05	0.050
		site	holobiont	2	0.02	1.69	0.131
		holobiont	time	1	0.01	1.39	0.214
		holobiont	site	1	0.01	1.53	0.186
	Host	time	site	3	0.01	12.28	**0.001**
		time	holobiont	3	0.01	11.92	**0.001**
		site	time	2	0.003	4.97	**0.002**
		site	holobiont	2	0.003	3.32	**0.015**
		holobiont	time	1	0.001	2.6	0.069
		holobiont	site	1	0.00	0.41	0.711
	Symbiont	time	site	3	0.24	19.35	**0.001**
		time	holobiont	3	0.24	19.40	**0.001**
		site	time	2	0.01	1.54	0.141
		site	holobiont	2	0.01	1.25	0.273
		holobiont	time	1	0.01	1.68	0.150
		holobiont	site	1	0.01	1.26	0.290

Porites	Combined	time	site	3	0.24	13.68	**0.001**
		time	holobiont	3	0.24	14.43	**0.001**
		site	time	2	0.13	11.53	**0.001**
		site	holobiont	2	0.06	4.64	**0.001**
		holobiont	time	1	0.14	25.94	**0.001**
		holobiont	site	1	0.07	10.18	**0.001**
	Host	time	site	3	0.06	19.65	**0.001**
		time	holobiont	3	0.06	19.20	**0.001**
		site	time	2	0.01	5.79	**0.001**
		site	holobiont	2	0.00	1.71	0.125
		holobiont	time	1	0.01	8.41	**0.002**
		holobiont	site	1	0.00	1.61	0.187
	Symbiont	time	site	3	0.17	15.06	**0.001**
		time	holobiont	3	0.17	15.45	**0.001**
		site	time	2	0.12	15.23	**0.001**
		site	holobiont	2	0.05	5.98	**0.001**
		holobiont	time	1	0.13	33.23	**0.001**
		holobiont	site	1	0.06	13.57	**0.001**

Table S12. Evaluation of random effects in univariate linear mixed effect model analysis of host responses. ANOVA-like analysis of random effects of linear mixed effect models on each response using single term deletions. LogLik = log likelihood; AIC = Akaike Information Criterion; LRT = likelihood ratio test; DF = degrees of freedom. All responses were log+1 transformed in linear mixed effect model analysis. Linear mixed effect models were conducted for each genus and included timepoint, site, and their interactions as main effects with colony nested within holobiont (for *Porites* and *Pocillopora* only) as random effects. Bold indicates P<0.05. CRE indicates copper reducing elements. AFDW indicates ash-free dry weight. Holobiont indicates host haplotype and associated symbionts.

Response	Genus	Random Effect	LogLik	AIC	LRT	DF	P-value
**HOST RESPONSES**							
Antioxidant Capacity (µmol CRE mg AFDW-1)	*Acropora*	colony	171.27	-316.53	3.60	1	0.058
	Pocillopora	colony:holobiont	288.63	-549.27	0.00	1	1.000
		holobiont	288.63	-549.27	0.00	1	1.000
	Porites	colony:holobiont	172.34	-316.69	21.25	1	**<0.001**
		holobiont	176.27	-324.54	13.40	1	**<0.001**
Host Soluble Protein (mg protein mg AFDW-1)	*Acropora*	colony	120.38	-214.76	0.50	1	0.480
	Pocillopora	colony:holobiont	220.47	-412.95	0.00	1	1.000
		holobiont	219.68	-411.35	1.59	1	0.207
	Porites	colony:holobiont	149.54	-271.09	6.66	1	**0.010**
		holobiont	146.16	-264.33	13.42	1	**<0.001**
Host Biomass (mg AFDW cm-2)	*Acropora*	colony	9.18	7.63	0.33	1	0.568
	Pocillopora	colony:holobiont	37.03	-46.06	3.32	1	0.069
		holobiont	38.69	-49.38	0.00	1	1.000
	Porites	colony:holobiont	-28.16	81.32	0.19	1	0.666
		holobiont	-28.07	84.14	0.00	1	1.000
Respiration (RD) (µmol O2 cm-1 h-1)	*Acropora*	colony	72.00	-118.00	0.16	1	0.690
	Pocillopora	colony:holobiont	136.76	-245.53	5.76	1	**0.016**
		holobiont	139.64	-251.28	0.00	1	1.000
	Porites	colony:holobiont	64.83	-101.66	0.00	1	1.000
		holobiont	64.83	-101.66	0.00	1	1.000
Calcification (µmol CaCO3 mg cm-2 h-1)	*Acropora*	colony	77.98	-129.08	0.88	1	0.348
	Pocillopora	colony:holobiont	78.08	-128.15	0.27	1	0.602
		holobiont	78.21	-128.42	0.00	1	1.000
	Porites	colony:holobiont	12.98	2.04	0.00	1	1.000
		holobiont	12.98	2.04	0.00	1	1.000

Table S13. Evaluation of random effects in univariate linear mixed effect model analysis of symbiont responses. ANOVA-like analysis of random effects of linear mixed effect models on each response using single term deletions. LogLik = log likelihood; AIC = Akaike Information Criterion; LRT = likelihood ratio test; DF = degrees of freedom. All responses were log+1 transformed in linear mixed effect model analysis. Linear mixed effect models were conducted for each genus and included timepoint, site, and their interactions as main effects with colony nested within holobiont (for *Porites* and *Pocillopora* only) as random effects. Bold indicates P<0.05. PAR indicates photosynthetically active irradiance. AFDW indicates ash-free dry weight. Holobiont indicates host haplotype and associated symbionts.

Response	Genus	Random Effect	LogLik	AIC	LRT	DF	P-value
**SYMBIONT RESPONSES**							
Symbiont Cell Density (cells mg AFDW-1)	*Acropora*	colony	-45.41	116.81	7.11E-14	1	1.000
	Pocillopora	colony:holobiont	-81.54	191.07	9.71	1	**0.002**
		holobiont	-76.68	181.36	0.00	1	1.000
	Porites	colony:holobiont	-50.42	128.84	0.09	1	0.760
		holobiont	-59.09	146.18	17.43	1	**<0.001**
Symbiont Biomass (mg AFDW cm-2)	*Acropora*	colony	40.98	-55.96	0.00	1	0.952
	Pocillopora	colony:holobiont	60.37	-92.75	3.48	1	0.062
		holobiont	52.79	-77.57	18.65	1	**<0.001**
	Porites	colony:holobiont	-63.92	155.83	1.20	1	0.273
		holobiont	-69.74	167.47	12.84	1	**<0.001**
Total Chlorophyll (chl a + chl c2) (µg chl mg AFDW-1)	*Acropora*	colony	-37.21	100.42	0.00	1	1.000
	Pocillopora	colony:holobiont	-6.41	40.81	0.00	1	1.000
		holobiont	-6.41	40.81	0.00	1	1.000
	Porites	colony:holobiont	-31.07	90.15	0.00	1	1.000
		holobiont	-31.07	90.15	-6.39E-14	1	1.000
Total Cell-Specific Chlorophyll (chl a + chl c2) (µg cell-1)	*Acropora*	colony	1001.20	-1976.4	1.03	1	0.311
	Pocillopora	colony:holobiont	1237.20	-2446.4	7.26	1	**0.007**
		holobiont	1240.80	-2453.7	0.00	1	1.000
	Porites	colony:holobiont	1362.40	-2696.8	0.00	1	1.000
		holobiont	1359.80	-2691.7	5.11	1	**0.024**
Symbiont : Host Biomass	*Acropora*	colony	123.24	-220.49	0.67	1	0.415
	Pocillopora	colony:holobiont	197.07	-366.14	0.00	1	1.000
		holobiont	183.97	-339.93	26.21	1	**<0.001**
	Porites	colony:holobiont	167.58	-307.16	10.93	1	**<0.001**
		holobiont	166.76	-305.53	12.56	1	**<0.001**

Maximal Photosynthesis (PMAX) (µmol O2 cm-1 h-1)	*Acropora*	colony	39.79	-53.75	4.84	1	**0.028**
	Pocillopora	colony:holobiont	94.64	-161.27	0.10	1	0.757
		holobiont	94.68	-161.36	0.01	1	0.931
	Porites	colony:holobiont	10.87	6.27	0.00	1	1.000
		holobiont	10.87	6.27	0.00	1	1.000
Apparent Quantum Yield (AQY)	*Acropora*	colony	479.22	-932.44	17.97	1	**<0.001**
	Pocillopora	colony:holobiont	673.54	-1319.1	0.32	1	0.571
		holobiont	673.70	-1319.4	0.00	1	1.000
	Porites	colony:holobiont	553.83	-1079.7	0.00	1	1.000
		holobiont	552.73	-1077.5	2.21	1	0.137
Saturating Irradiance (IK) (PAR)	*Acropora*	colony	-39.41	104.82	6.87	1	**0.009**
	Pocillopora	colony:holobiont	-49.66	127.31	0.04	1	0.840
		holobiont	-49.63	127.27	0.00	1	1.000
	Porites	colony:holobiont	-56.80	141.6	0.00	1	1.000
		holobiont	-57.85	143.7	2.10	1	0.147
Compensation Irradiance (IC) (PAR)	*Acropora*	colony	-65.84	157.69	0.01	1	0.918
	Pocillopora	colony:holobiont	-84.79	197.59	0.04	1	0.843
		holobiont	-84.77	197.55	0.00	1	0.967
	Porites	colony:holobiont	-67.02	162.04	0.00	1	0.954
		holobiont	-71.99	171.97	9.93	1	**0.002**

Table S14. Redundancy analysis of variance constrained in symbiont and host physiology by environmental characteristics. P-values of each term from redundancy analysis (RDA) and model analysis of variance constrained in host and symbiont of each genus by host and symbiont responses with holobiont identity included in models for each biological level. Main effects were mean light (solar radiance in kWh m-2) and mean temperature (°C) that were scaled for analysis. Gray boxes indicate no significant effect. Bold indicates P<0.05.

Biological Level	Genus	Main Effect	DF	Variance	F	P-value
Host Responses	*Acropora*	light	1	0.66	13.04	**0.001**
		temperature	1	0.27	5.41	**0.002**
	Pocillopora	light	1	0.01	9.73	**0.001**
		temperature	1	0.01	19.00	**0.001**
	Porites	light	1	0.01	4.18	**0.007**
		temperature	1	0.05	30.58	**0.001**
Symbiont Responses	*Acropora*	light	1	0.30	52.63	**0.001**
		temperature	1	0.11	18.75	**0.001**
	Pocillopora	light	1	0.19	38.58	**0.001**
		temperature	1	0.06	12.04	**0.001**
	Porites	light	1	0.11	20.67	**0.001**
		temperature	1	0.05	9.17	**0.001**

Table S15. Redundancy analysis of variance constrained in symbiont ITS2 communities by host and symbiont responses. Distance-based RDA analysis of variance constrained in symbiont communities of each species by host and symbiont responses (Fig 2). Variance constrained by host and symbiont responses on symbiont communities in each species expresed as a percentage of variance in symbiont ITS2 community explained by significant models. Bold indicates $P<0.05$. DF = degrees of freedom/number of responses tested at each biological level; SS = sum of squares.

Genus	(Biological	DF	SS	F value	P-value	Community	Community by Model
Acropora	Host	5	0.38	0.64	0.751		
	Symbiont	9	0.90	0.84	0.624		
Pocillopora	Host	5	1.29	0.65	0.886	2.64	0
	Symbiont	9	6.88	2.11	**0.001**	14.09	7.43
Porites	Host	5	6.68	4.26	**0.001**	14.17	10.84
	Symbiont	9	10.66	4.05	**0.001**	22.58	17.01

Table S16. Redundancy analysis of variance constrained in symbiont ITS2 communities by host and symbiont responses at the genus level. P-values of each term from distance-based RDA (dbRDA) analysis of variance constrained in symbiont communities of each genus by host and symbiont responses with holobiont identity included in models for each biological level. Significance of variance explained in symbiont communities by each host and symbiont response as analyzed with dbRDA models. Gray boxes indicate no significant effect. Bold indicates P<0.05.

	Acropora	*Pocillopora*	*Porites*
**Host Responses**			
Antioxidant capacity	0.277	0.992	**0.001**
Host biomass	0.366	0.791	**0.002**
Host protein	0.878	0.826	0.077
Respiration	0.543	0.096	0.428
Calcification	0.671	0.826	0.269
**Symbiont Responses**			
Cell density	0.145	0.514	**0.002**
Symbiont biomass	0.512	**0.014**	**0.001**
Total chlorophyll	0.684	**0.001**	0.423
Total chlorophyll per cell	0.402	**0.046**	0.101
PMAX	0.832	0.921	**0.038**
AQY	0.645	0.757	**0.002**
IK	0.235	0.254	0.354
IC	0.159	**0.024**	0.214
S:H biomass	0.767	**0.033**	0.438
